# Targeted long-read eukaryotic profiling of human stool by broad-range 18S–28S rRNA gene amplicon sequencing on Oxford Nanopore Technologies: a proof-of-concept study

**DOI:** 10.3389/fmicb.2026.1904425

**Published:** 2026-09-30

**Authors:** Ruslan Kalendar, Asset Daniyarov, Ulykbek Kairov, Samat Kozhakhmetov, Dauren Yerezhepov, Bakytgul Yermekbayeva, Dos Sarbassov, Almagul Kushugulova

**Affiliations:** 1 Helsinki Institute of Life Science (HiLIFE), University of Helsinki, Helsinki, Finland; 2 Center for Life Sciences, National Laboratory Astana, Nazarbayev University, Astana, Kazakhstan; 3 Faculty of Natural Sciences, L.N. Gumilyov Eurasian National University, Astana, Kazakhstan

**Keywords:** 18S rRNA, 28S rRNA, Blastocystis hominis, eukaryotic metagenomics, gut mycobiome, long-read amplicon sequencing, Malassezia, Oxford Nanopore Technologies

## Abstract

The gut eukaryome, comprising the protists and fungi that fall within the strict definition of the microbiota together with the macroscopic helminths and arthropods that lie outside it, is under-detected by clinical microscopy and by short-read 16S/18S sequencing, yet is increasingly implicated in inflammatory and autoimmune disorders, including psoriasis. Existing 18S V4/V9 primers either lack cross-kingdom universality or fail to discriminate closely related species. We designed and evaluated broad-range PCR primers anchored in conserved blocks of nuclear 18S and 28S ribosomal genes, flanking a 1.0–3.5 kb cassette spanning the 3′-end of 18S, ITS1, 5.8S, ITS2, and the 5′-end of 28S. Stool samples from 91 plaque-psoriasis patients and 92 matched controls were collected; a subset of 32 samples was profiled by Oxford Nanopore amplicon sequencing using the EPI2ME wf-metagenomics pipeline (Kraken + Bracken) against the NCBI core_nt database. The gut eukaryome was dominated by Ascomycota and Protista, with 51 species-level entries assigned to *Candida* (37 named binomials), 27 entries assigned to *Blastocystis* (12 subtype-labelled entries), 20 entries assigned to Malassezia (15 named species), and 37 entries assigned to *Cladosporium* recovered from a single PCR tube. *Blastocystis hominis* was detected in 37.5% of subjects (≥50-read threshold; mean 7.5% in carriers), with concurrent detection of 12 subtype-labelled entries (ST1-ST4, ST7, ST10-ST15, ST24) and 11 named isolate entries. Psoriasis-relevant *Candida* species, such as *C. albicans* (34.4%), *C. tropicalis* (34.4%), *C. parapsilosis* (31.2%), and *Malassezia restricta* (9.4%; up to 12.1%) were co-detected. Long-read sequencing of a broad-range 18S -28S rRNA gene cassette provides a single-tube workflow for the simultaneous recovery of fungal, protistan, and plant-derived eukaryotic sequences from human stool.

## 1 Introduction

The human gastrointestinal tract harbours a complex polymicrobial community, whose bacterial component has been mapped at unprecedented resolution by 16S rRNA gene sequencing and shotgun metagenomics. In contrast, the eukaryotic components of the gut microbiome, collectively termed the eukaryome, remain poorly characterised. The eukaryome comprises unicellular protists and fungi, which fall within the strict definition of the microbiota, together with helminths and arthropods, which are macroscopic and therefore lie outside that definition (Berg et al., 2020); the term is used here in this inclusive, operational sense. These organisms remain under-characterized despite mounting evidence that they modulate host immunity, intestinal barrier integrity, and the trajectory of immune-mediated diseases (Lukes et al., 2015; Laforest-Lapointe and Arrieta, 2018). Population-scale studies estimate that the protist *Blastocystis* alone colonises ≥ 1 billion people worldwide (Piperni et al., 2024); however, its clinical significance is debated, and its detection in routine stool diagnostics is unreliable.

Psoriasis is a chronic, immune-mediated, non-communicable inflammatory dermatosis that affects 2–4% of the global population and is increasingly recognised as a systemic disease with extra-cutaneous involvement (Griffiths et al., 2021). Plaque psoriasis is used here solely as the clinical setting in which the workflow is demonstrated; the aim of this study is methodological, and the clinical background summarised in this paragraph serves only to explain this choice. The pathogenesis of psoriasis involves dysregulated Th17/Th22 axes and a maladaptive interplay between skin-resident immunity and the gut-skin axis. Cohort studies have linked psoriasis to perturbations of the gut bacteriome (decreased *Akkermansia muciniphila* and increased Firmicutes/Bacteroidetes ratio), but the contribution of the gut eukaryome, in particular of *Blastocystis*, *Candida*, and *Malassezia* species, has not been systematically explored. Anecdotal reports suggest that clearance of *Blastocystis* can coincide with remission of atopic dermatitis and plaque psoriasis (Andersen and Stensvold, 2016), but such observations have not been tested in controlled studies. Evidence-based assessment of eukaryotic carriage therefore requires a sensitive, standardised molecular assay, which the present study sets out to provide.

DNA metabarcoding has revolutionised eukaryotic community ecology and microbial diagnostics. The nuclear ribosomal RNA operon comprising 18S rRNA gene (small ribosomal subunit, SSU), the internal transcribed spacers ITS1 and ITS2 flanking the 5.8S rRNA gene, and the 28S rRNA gene (large ribosomal subunit, LSU) is the canonical target because it combines highly conserved blocks suitable for universal primer placement with rapidly evolving variable domains that resolve genus- and species-level taxa (Guillou et al., 2013; Quast et al., 2013; Hadziavdic et al., 2014; Del Campo et al., 2019). Most existing eukaryote metabarcoding studies, however, target only the V4 or V9 hypervariable region of the 18S rRNA gene, producing fragments of 250–500 bp that are well-suited to short-read Illumina platforms but offer limited phylogenetic resolution and frequently fail to discriminate closely related *Candida*, *Malassezia*, or *Blastocystis* subtypes (Pawlowski et al., 2012). Fungi are typically profiled with a separate ITS1 or ITS2 amplicon, whereas protists may require taxon-specific primers, multiplying the number of laboratory workflows and complicating quantitative comparison across kingdoms. Long-read sequencing on Oxford Nanopore Technologies (ONT) platforms MinION, GridION, and PromethION now enable single-molecule reads spanning the full 6–8 kb rRNA gene operon and is increasingly applied to microbial diagnostics, full-length 16S profiling, and metabarcoding of eukaryotic environmental DNA (Jain et al., 2016; Sereika et al., 2022). Compared to short-read approaches, ONT amplicon sequencing offers (i) species- and even strain-level taxonomic resolution from a single PCR product, (ii) library preparation in under 15 min using rapid transposase-based barcoding, (iii) 96-sample multiplexing per flow cell, and (iv) real-time data acquisition that supports run termination once sufficient coverage is achieved. The principal historical drawback high per-base error has been substantially mitigated by R10.4 chemistries, super-accuracy basecalling models, and consensus-based polishing, with modal raw-read accuracies now exceeding 99% (Sereika et al., 2022). Despite these technological advances, very few universal eukaryote primer sets have been specifically designed and benchmarked for ONT long-read amplicon sequencing of human stool. The ideal primer pair must (i) match conserved sites with no 3′-terminal mismatches across all eukaryotic kingdoms; (ii) avoid degenerate bases that broaden the amplification window unpredictably; (iii) yield amplicons short enough to amplify efficiently from low-input, fragmented stool DNA (≤ 3.5 kb) yet long enough to span multiple variable regions for robust taxonomy; and (iv) selectively exclude or under-represent host (human) DNA to preserve sequencing depth for the microbial fraction.

Here, we report the rational design, *in silico* evaluation, and first clinical application of a panel of broad-range 18S–28S rRNA gene primer pairs developed against these criteria. This study is a methodological proof of concept: it establishes that the cassette can be amplified from stool in a single tube and sequenced on ONT, and it characterizes the analytical behaviour of the resulting data. It does not validate quantitative performance against defined mock communities, nor does it seek to identify psoriasis-associated eukaryotic signatures. We applied the optimised assay to stool samples from a clinical cohort of patients with plaque psoriasis and matched healthy controls from Kazakhstan, generated long-read ONT amplicon datasets, and developed a Dorado-minimap2-Kraken2 bioinformatic pipeline for taxonomic profiling of the gut eukaryome. We show that the assay simultaneously detects *Blastocystis*, commensal, and opportunistic yeasts, and dietary plant DNA from a single tube reaction, providing a candidate framework for clinical and population-scale eukaryome surveillance that now requires formal analytical validation.

## 2 Materials and methods

### 2.1 Study design, ethics, and participant recruitment

This work was conducted in two sequential phases. Phase I (cross-sectional clinical phase, January 2017; December 2018) recruited 91 patients with histologically confirmed mild-to-moderate plaque psoriasis (vulgaris) in the progressive stage and 92 age-, sex- and locality-matched healthy adult volunteers from the Department of Dermatovenereology of Astana Medical University and the Center of Dermatology and Prevention of Sexually Transmitted Diseases, Astana, Kazakhstan. Phase II (prospective metagenomic and immunological phase) randomly selected 20 psoriasis patients and 20 controls from the Phase I cohort for stool eukaryome profiling; of these 40 samples, 32 yielded high-quality long-read amplicon datasets and were retained for downstream analysis. The eight excluded libraries did not meet the pre-defined quality criteria given below. The composition of the final sequencing set (barcodes BC39-BC70) group assignment, sex, age, sampling time point relative to treatment, and any intervention received is given in Supplementary Table S2, and the recruitment-to-analysis flow is summarised in Supplementary Figure S5. Because this subset is small and not balanced by design, no formal case–control comparison was attempted. Inclusion criteria for the patient group were confirmed plaque psoriasis (vulgaris) of mild-to-moderate severity, age 30–45 years, and uninterrupted residence in the same locality for the preceding 5 years. Exclusion criteria comprised: any other chronic dermatosis or gastrointestinal disorder; documented dairy allergy; major comorbidities (renal, hepatic, cardiovascular, pulmonary, oncological, psychiatric, or decompensated endocrine disease, tuberculosis, or HIV infection); pregnancy or lactation; participation in another clinical trial within the preceding 3 months; and antibiotic use within the preceding 3 months. All participants received standard topical and systemic management for psoriasis (desensitising, antihistamine, and topical agents; physiotherapy). A subset of 20 patients received, in addition to standard therapy, lyophilized mare’s milk (“Saumal”) at 60 mg/day for 12 weeks. Disease severity was monitored by the Psoriasis Area and Severity Index (PASI) at baseline, week 12, and at 6-month outpatient follow-up. The study was approved by the Local Bioethics Committee of Astana Medical University and the National Laboratory Astana, Nazarbayev University (IORG0006963, Protocol No. 05–2020). Phase I recruitment and stool collection (2017–2018) were covered by an earlier approval of the Local Bioethics Committee of the University Medical Center, Astana, Kazakhstan (Protocol No. 07–2017); the metagenomic work reported here was performed under Protocol No. 05–2020. Both approvals are stated in the Declarations. Written informed consent was obtained from every participant prior to enrolment. The study was conducted in accordance with the Declaration of Helsinki.

### 2.2 Stool collection, DNA extraction, and quality control

Fresh stool was collected immediately post-defecation directly into DNA/RNA Shield Collection Tubes (Zymo Research, R1101, Irvine, CA, USA), homogenised by inversion, and stored at +4 °C until extraction (≤ 7 days). Total genomic DNA was extracted from approximately 200 mg of stool using the ZymoBIOMICS DNA Miniprep Kit (Zymo Research, D4300) following the manufacturer’s protocol with the supplied bead-beating step (BashingBead lysis tubes, 10 min, maximum speed) to ensure efficient lysis of yeasts, helminth eggs, and *Blastocystis* cysts. Sterile molecular biology-grade water served as a parallel negative extraction control in every batch. DNA integrity was verified by 1.0% (w/v) agarose gel electrophoresis. Concentration and purity were determined using a NanoDrop One (Thermo Fisher Scientific, Waltham, MA, USA), accepting samples with an A_260_/A_230_ ratio of approximately 2.0, and a Qubit 3.0 dsDNA Broad Range assay (Invitrogen, Carlsbad, CA, USA). Eluates were normalised to 25 ng/μL and stored at −80 °C in single-use aliquots. Quality criteria were defined *a priori* and applied at two levels. A DNA extract was accepted for PCR if it showed a discrete high-molecular-weight band on the integrity gel, an A_260_/A_230_ ratio of approximately 2.0, and a concentration sufficient to provide 25 ng of template per reaction. A sequencing library was retained for taxonomic analysis if, after demultiplexing and after the MinKNOW quality and length filters (Q ≥ 9; ≥ 200 bp), the number of classified reads exceeded the contamination background defined by the negative controls processed in the same batch.

### 2.3 Design of universal eukaryotic 18S and 28S primers

Reference 18S and 28S rRNA gene sequences were retrieved from NCBI GenBank, including both individual gene submissions and complete genome assemblies, for representative species across all major eukaryotic kingdoms (SAR Stramenopiles, Alveolata, Rhizaria; Amoebozoa; Metamonada; Percolozoa; Fungi Ascomycota, Basidiomycota, Mucoromycota; Viridiplantae; Plantae sensu stricto; Animalia). Sequence quality was filtered by reciprocal multiple alignment of con-specific and con-generic accessions (Adl et al., 2019); only complete, full-length, and unambiguously identified entries were retained. GenBank rather than RefSeq was used as the source of reference sequences because RefSeq contains comparatively few curated, full-length nuclear rRNA gene cassettes for protists, early-diverging fungi and helminths, whereas the corresponding regions are available in GenBank as directly submitted rRNA gene records or within whole-genome assemblies. Because primer design requires the contiguous 18S-ITS-28S region rather than annotated gene models, completeness of the operon was prioritised over curation status; every retained accession was nevertheless checked manually for annotation consistency and cross-checked against con-generic sequences. Reference species were selected for phylogenetic spread and for the availability of a complete 18S-28S cassette within a single record, not for clinical relevance. Multiple sequence alignment of the curated dataset was performed using MAFFT v7.520 with the L-INS-i algorithm (Katoh and Standley, 2013).

Conserved blocks were identified visually by multiple alignments. Candidate primer sites were selected to satisfy all of the following criteria: (i) zero mismatches at the 3′-terminus across all reference sequences within each kingdom; (ii) at most one or two transitions tolerated within the 5′-half of the primer (which empirically does not abolish PCR efficiency); (iii) no internal degenerate positions; (iv) calculated melting temperature ≥ 65 °C, compatible with two-step PCR using combined annealing and extension at 68–72 °C; (v) GC content 45–65%; and (vi) linguistic complexity ≥ 70% to minimise the formation of secondary structures and primer-dimers. Primer pair geometry was constrained to yield amplicons of 1.0–3.5 kb across most eukaryotic taxa, simultaneously enabling efficient long-range PCR from low-input stool DNA and biasing against amplification of full-length mammalian (host) cassettes that exceed 5 kb. Candidate primers were exhaustively tested *in silico* with FastPCR (Kalendar et al., 2024; Kalendar, 2025) against every reference sequence in the curated database. Pairs producing 100% primer template homology and amplicons within the target size window across all kingdom-level references were retained for experimental validation. This *in silico* evaluation was performed against the curated kingdom-representative panel assembled for primer design, and not against the complete NCBI nucleotide collection. Therefore, it establishes predicted coverage of the targeted eukaryotic lineages but does not exhaustively enumerate potential off-target templates, in particular prokaryotic 16S–23S rRNA gene regions, which contain the same universally conserved motifs. The complete list of primers, their thermodynamic parameters, and binding coordinates on representative reference sequences are provided in Table 1; full kingdom × primer-pair amplicon-size matrices are provided in Supplementary Table S1.

**Table 1:** The universal eukaryotic 18S and 28S rRNA primers used in this study, with thermodynamic properties and binding coordinates on eight reference species spanning eight phyla and four eukaryotic supergroups.

Primer ID	Sequence (5′ → 3′)	nt	Tm (°C)	GC (%)	LC (%)	*Saccharomyces cerevisiae* (CP033498) Ascomycota (fungi)	*Gymnodinium catenatum (DQ785882) Dinoflagellata (dinoflagellates)*	*Blastocystis hominis* (KF002541) Bigyra (stramenopiles)	*Plasmodium falciparum* (LR131487) Apicomplexa (apicomplexans)	*Giardia muris* (X65063) Fornicata (diplomonads)	*Adineta vaga* (GQ398061) Rotifera (bdelloid rotifer)	*Litomosoides sigmodontis* OY718804 (chr 2) Nematoda (filarial nematode)	*Marchantia polymorpha* (AB021684) Marchantiophyta (liverwort)
18S rRNA gene forward primers													
5780	TGTGAAAGTAGATCGTGATGGGGATTGATGC	31	68.4	45.2	76	–	–	1489 → 1519	–	–	–	–	–
5781	CCTAGTAAACGCAAGTCATCAACTTGCAT	29	65.7	41.4	83	–	–	1549 → 1577	–	–	–	–	–
5728	CTGCGGCTTAATTTGACTCAACACGG	26	67.0	50.0	91	2553 → 2578	1782 → 1807	1149 → 1174	1119 → 1144	4357 → 4378*	5338 → 5363*	2095 → 2120	1600 → 1625
5751	CCTGCCCTTTGTACACACCGCC	22	69.3	63.6	78	2998 → 3019	2231 → 2252	1588 → 1609	1760 → 1781	4238 → 4259*	5787 → 5808*	2545 → 2566	2050 → 2071
5753	CCCTTTGTACACACCGCCCGTC	22	68.9	63.6	78	3002 → 3023	2235 → 2256	1592 → 1613	1764 → 1785	4242 → 4263	5791 → 5812	2549 → 2570	2054 → 2075
5763	TAGAGGAAGGAGAAGTCGTAACAAGGT	27	65.0	44.4	69	3122 → 3148*	2352 → 2378	1714 → 1740	1879 → 1905	4334 → 4360	5915 → 5941*	2672 → 2698*	2176 → 2202
5390	AGGTTTCCGTAGGTGAACCTGC	22	65.4	54.5	90	3145 → 3166	2375 → 2396	1737 → 1758	1902 → 1923	4357 → 4378	5938 → 5959*	2695 → 2716	2199 → 2220
28S rRNA gene reverse primers													
5765	CAAAGTCCTTTTCATCTTTCCCTCGCGG	28	68.3	50.0	78	–	–	–	–	–	–	–	4192 ← 4219
5394	CCTTGGTCCGTGTTTCAAGACG	22	64.5	54.5	95	4568 ← 4589	3724 ← 3745	UN	3369 ← 3390	–	7504 ← 7525*	4601 ← 4622	4499 ← 4520
5647	TTCGATTAGTCTTTCGCCCCTA	22	61.4	45.5	80	4835 ← 4856	3978 ← 3999	UN	3678 ← 3699	5222 ← 5243	7812 ← 7833*	4915 ← 4936	4768 ← 4789
5730	TGGTTCGATTAGTCTTTCGCCCCTA	25	65.9	48.0	86	4835 ← 4859	3978 ← 4002	UN	3678 ← 3702*	5222 ← 5246*	7812 ← 7836*	4915 ← 4939	4768 ← 4792
5754	GGTGAGTTGTTACACACTCCTTAGC	25	63.7	48.0	84	5186 ← 5210	4325 ← 4349	UN	4038 ← 4062	5560 ← 5584*	8169 ← 8193*	5275 ← 5299	5122 ← 5146
5758	GAGAGTCATAGTTACTCCCGCCGTTTACC	29	68.3	51.7	81	6169 ← 6197	5292 ← 5320*	UN	5185 ← 5213	6327 ← 6355	9243 ← 9271	6406 ← 6434	6142 ← 6170
5759	GAGTCATAGTTACTCCCGCCG	21	62.9	57.1	87	6175 ← 6195	5298 ← 5318*	UN	5191 ← 5211	6333 ← 6353	9249 ← 9269	6412 ← 6432	6148 ← 6168

LC, linguistic complexity. *Mismatch at the 5′-end of the primer that does not abolish PCR efficiency. Numbers in primer-binding columns are 1-based coordinates on the cited reference; arrows denote orientation. UN - the primer locations are unknown, as complete 28S rRNA sequences are not available. In plants and many protists the nuclear large-subunit rRNA gene is designated 25S or 26S rather than 28S; these are homologous genes, and the reverse primers anchor in a block conserved across all of them. Accession AB021684 is annotated accordingly. Litomosoides sigmodontis is included as a representative filarial nematode rather than as a gut-dwelling helminth. Primer 5,765 is Viridiplantae-selective and is used with the forward primer 5751.

### 2.4 PCR amplification

PCR was performed in 25 μL reactions containing 25 ng template DNA, 1 × Phusion HF Buffer (with 1.5 mM MgCl_2_), 0.2 mM each dNTP, 0.2 μM each primer, and 1 U Phusion Hot Start II High-Fidelity DNA Polymerase (Thermo Fisher Scientific). Thermal cycling on a SimpliAmp Thermal Cycler (Thermo Fisher Scientific) was: initial denaturation 98 °C / 90 s; 32 cycles of 98 °C / 5 s and 72 °C / 120 s (combined annealing and extension); final extension 72 °C / 1 min. The principal primer pair used for clinical samples was 5753–5730 (Table 1), yielding an estimated 1.0–3.4 kb amplicon across the eukaryotic kingdoms tested. PCR products were resolved on 1.5% (w/v) agarose gels in 1 × TAE at 90 V for 3 h, stained with ethidium bromide (0.5 μg/mL), and imaged on a Bio-Rad ChemiDoc XRS + system using a Thermo Scientific GeneRuler DNA Ladder Mix (100–10,000 bp; SM0332) for size reference. Confirmatory Sanger sequencing was performed on gel-purified single-band products using an ABI3700 capillary sequencer (Thermo Fisher Scientific) with BigDye Terminator v3.1 chemistry.

For targeted detection of Blastocystis in stool DNA, a second, genus-selective assay was used: forward primer 5,780, which anchors in an 18S rRNA gene block conserved within *Blastocystis* but not across eukaryotes, was paired with the broad-range eukaryotic 28S reverse primer 5730 (Table 1). This combination yields an amplicon of approximately 1.3 kb, measured on agarose gels and confirmed by Sanger sequencing. The binding site of 5730 in *Blastocystis* could not be assigned *in silico* and is given as UN in Table 1, because complete 28S rRNA gene sequences are not available for this genus. The genus-selective assay was used only for confirmatory identification; all barcoded sequencing libraries were generated with the broad-range pair 5753/5730 described above. Amplicons for Sanger sequencing were generated from human faecal DNA with the 5728/5730 primer pair, separated on 1% agarose, and individual bands were excised and gel-purified before capillary sequencing. Consensus sequences of the 16 individually sequenced amplicons, the corresponding electropherograms, and the laboratory notebook records of the amplification and gel-purification steps are provided in Supplementary Data S1; per-amplicon identifications are summarised in Supplementary Table S6.

A second lineage-selective configuration targets Viridiplantae. The reverse primer 5765 anchors in a 28S rRNA gene block conserved among green plants; paired with the broad-range 18S forward primer 5751 it is predicted to give a product of 2,170 bp on the *Marchantia polymorpha* reference (AB021684). Because 5765 matched none of the seven non-plant references examined here (Table 1), the 5751/5765 combination is expected to amplify plant templates selectively from mixed-template DNA in which fungal, animal and arthropod sequences are simultaneously present the situation in stool. This configuration was not used to generate the barcoded libraries reported here; it is described as a targeted option for studies in which dietary or environmental plant DNA is the analytical focus. In a side-by-side comparison on 12 plant DNA extracts, the 5765/5751 combination yielded a single product of uniform size, whereas the broad-range 5756/5751 combination yielded multiple products from the same templates (Supplementary Data S1, January 2024).

### 2.5 Multiplex long-range PCR panel

To broaden taxonomic coverage in a single tube, six primers from Table 1, four 18S forward primers (5390, 5751, 5753, 5763) and two 28S reverse primers (5394, 5730), were combined into a single multiplex reaction. PCR was performed in 25 μL reactions containing 25 ng template DNA, 1 × Phusion HF Buffer (with 1.5 mM MgCl_2_), 0.2 mM each dNTP, 20 nM each primer (6 primers panel: 5390, 5394, 5730, 5751, 5753, 5763), and 1 U Phusion Hot Start II High-Fidelity DNA Polymerase (Thermo Fisher Scientific). Thermal cycling on a SimpliAmp Thermal Cycler (Thermo Fisher Scientific) was performed as follows: initial denaturation at 98 °C / 90 s; 32 cycles of 98 °C / 5 s and 72 °C / 120 s (combined annealing and extension); and final extension at 72 °C / 1 min.

### 2.6 ONT library preparation and sequencing

PCR products from replicate reactions performed on the same stool sample were pooled; however, DNA or PCR products from different individuals were never pooled. One hundred nanograms of each per-sample pool was purified with 0.6 × AMPure XP beads (Beckman Coulter), eluted in 10 μL nuclease-free water, and quantified by Qubit. Sequencing libraries were prepared using the Rapid Barcoding Kit 96 (SQK-RBK110-96, Oxford Nanopore Technologies) according to the manufacturer’s protocol. Each batch included one no-template extraction control and one PCR no-template control. Barcoded libraries were pooled equimolarly and loaded onto a FLO-MIN106 R9.4.1 flow cell on a GridION Mk1. Sequencing was run for 25 h with the following MinKNOW v23.11.7 settings: live high-accuracy basecalling at 450 bps (Dorado v7.2.13), minimum quality score 9, minimum read length 200 bp, active channel selection on, pore scan frequency 1.5 h, reserved pores on, mid-read barcode filtering on (minimum barcode score 60, mid-read score 50), read splitting off, modified basecalling off, barcode trimming off. Raw data were output as compressed FAST5 (vbz) and FASTQ (gzip) at 4,000 reads per file (MinKNOW Core v5.8.6; Bream v7.8.2; Configuration v5.8.6).

### 2.7 Bioinformatic analysis

The 32-sample cohort was subsequently processed using the EPI2ME wf-metagenomics v2.14.2 Nextflow workflow (Oxford Nanopore Technologies), which implements taxonomic classification with Kraken 2.1.3 (Wood et al., 2019) and read-level abundance re-estimation with Bracken v3.0.1 (Lu et al., 2022). Classification was performed using the NCBI core_nt nucleotide database (release October 2025), which incorporates a substantially expanded reference coverage for eukaryotic rRNA genes compared to earlier builds. The following pipeline parameters were applied: --kraken2_confidence 0.1, −-bracken_threshold 10, −-taxonomic_rank S, −-min_len 200, −-min_read_qual 10, −-abundance_threshold 0.01, and--threads 16. Reads were aligned using minimap2 v2.28 (Li, 2018) and processed using Samtools v1.21 (Nilsson et al., 2019).

An eukaryote-wide reference (core_nt) was preferred over a fungus- or protist-restricted database for three reasons. First, the amplified cassette is pan-eukaryotic, so a restricted reference would force reads from non-target eukaryotes dietary plants and animals, environmental fungi, host DNA either to remain unclassified or to be placed on the nearest available fungal or protistan reference, artificially inflating apparent target diversity. Second, retaining the full eukaryotic reference space makes off-target and dietary signal explicit and quantifiable rather than hidden. Third, the conserved 18S and 28S flanks of the cassette are shared across kingdoms, so k-mer profiles overlap substantially between distantly related eukaryotes; Kraken2 resolves this ambiguity by assigning a read to the lowest common ancestor of all matching references, with the consequence that reads carrying little information beyond the conserved core are placed at high taxonomic ranks or on “uncultured” and “environmental sample” entries rather than being confidently mis-assigned to a species. Non-target assignments were interpreted accordingly: reads assigned to *Viridiplantae* and to edible *Metazoa* were treated as biological signal of dietary origin; reads assigned to *Homo sapiens* and to other primates as residual host amplification; and reads assigned to taxa with no plausible route into the human gut, or to synthetic constructs, as classification artifacts. Reads assigned to Bacteria, Archaea and Viruses which may arise from off-target amplification of co-extracted genomic DNA, from k-mer cross-mapping of conserved rRNA gene regions, or from both were excluded prior to all downstream analyses, and the corresponding counts are reported in Results. Residual host (*Homo sapiens*) reads and reads assigned to non-human primates (*Macaca mulatta*, *M. fascicularis*, *M. nemestrina*) were likewise removed. Species-level entries were flagged as probable classification artifacts, rather than as true low-abundance taxa, when they met at least two of the following criteria: (i) a prevalence pattern incompatible with biological signal, namely detection in all or nearly all samples at uniformly low relative abundance, which is the pattern expected from systematic k-mer cross-mapping; (ii) an ecological origin with no plausible route into the human gut under the sampling conditions, such as obligate marine or freshwater hosts, obligate pathogens of other mammals, or laboratory model organisms; (iii) assignment to a reference whose conserved rRNA gene region is closely similar to that of an abundant taxon detected in the same sample; and (iv) synthetic or vector-derived construct sequences. This filter is a heuristic: the underlying reads were not verified at sequence level, and some of them may derive from genuine off-target amplification rather than from database cross-mapping. Read counts, prevalence and abundance profiles of the removed entries are given in Supplementary Table S4. On this basis five entries were excluded: *Photobacterium leiognathi* (100% prevalence indicating systematic k-mer misclassification), *Poeciliopsis monacha* (freshwater fish), *Mycoplasmopsis bovis* (obligate bovine pathogen), *Arabidopsis thaliana* (model plant), and synthetic construct sequences. After filtering, 687 eukaryotic species-level entries were retained. All diversity metrics and prevalence estimates reported herein are computed exclusively from the eukaryotic fraction.

Alpha diversity (observed species richness, Shannon H, Simpson 1-D) was computed from Bracken-corrected species-level abundance tables in Python 3.9 (pandas v2.3.3, SciPy v1.9). Beta diversity was assessed by Bray–Curtis dissimilarity with principal coordinates analysis (scikit-learn v1.1). Minimum read-count thresholds were applied for prevalence reporting: ≥50 reads for *Blastocystis* and *Candida*, ≥10 reads for *Malassezia*, calibrated against negative-control distributions (median 28 reads, max 82). Because Bracken output was filtered at 10 reads per taxon per sample, every taxon-sample observation reported here is supported by at least 10 assigned reads; read-level singletons are consequently unavailable, and coverage estimators of the Good or Chao type could not be applied to these data. Sensitivity of the diversity and core-community estimates to sequencing-depth heterogeneity was therefore assessed by rarefaction and by exclusion of shallow libraries: every sample was rarefied without replacement to common depths of 909, 3,000, 5,000 and 10,000 classified eukaryotic reads (50 random subsamplings per depth, samples below the target depth being omitted at that depth); prevalence, sparsity and core membership were recomputed after excluding samples below 3,000 and below 5,000 reads; and the Spearman rank correlation between library size and observed richness was computed before and after rarefaction (Chao and Jost, 2012). Genus-level prevalence was additionally recomputed using relative-abundance thresholds (≥ 0.1% and ≥ 1% of the eukaryotic fraction) in place of absolute read thresholds. Analyses were performed in Python 3.9 with NumPy, pandas, SciPy and scikit-learn; scripts are available in the repository cited under Availability of data and materials.

## 3 Results

### 3.1 Universal 18S–28S primer set design and in silico validation

Iterative analysis of the curated multi-kingdom rRNA gene alignment identified seven conserved blocks within the 18S gene and seven within the 28S gene that were suitable for primer placement under our design constraints (Table 1). The selected forward primers (5780, 5781, 5728, 5751, 5753, 5763, 5390) target progressively more 3′-ward sites within 18S, from the V4–V5 boundary to the 18S–ITS1 junction (5390), while the reverse primers (5765, 5394, 5647, 5730, 5754, 5758, 5759) walk along 28S from the D1 expansion segment (5394) to the D2–D3 region (5759). All 14 oligonucleotides have melting temperatures of 61–70 °C, GC content 44–64%, and linguistic complexity 69–98%, fulfilling our two-step PCR design specification.

Combinatorial *in silico* PCR across the curated kingdom-representative panel demonstrated that the pair 5753 (forward, 18S) and 5730 (reverse, 28S) provides the most balanced compromise between (i) universal eukaryotic coverage, (ii) host-DNA exclusion, and (iii) ONT-compatible amplicon size (Table 2; Supplementary Table S1). Predicted amplicon lengths for this pair are given in Table 2 and range from about 1.0 kb in Metamonada to 1.5–2.5 kb across fungi, protists, plants and invertebrates, and about 4.2 kb for *Homo sapiens*, confirming that the human (host) cassette lies at or beyond the upper boundary of efficient long-range PCR and is therefore strongly under-represented in the final library. The reported values were as follows: 1,005 bp for *Giardia muris*; 1,475 bp for *Naegleria fowleri*; 1,593 bp for *Saccharomyces cerevisiae*; 1,939 bp for *Plasmodium falciparum*; 2,046 bp for *Adineta vaga*; 2,199 bp for *Danio rerio*; 2,363 bp for *Polysphondylium pallidum*; 2,460 bp for *Xenopus laevis*; 2,460 bp *for Drosophila melanogaster*; and 4,212 bp for *Homo sapiens*. These values confirm that human (host) DNA generates an amplicon at the upper boundary of efficient long-range PCR and is therefore strongly under-represented in the final library. Alternative primer pairs that retain 100% 3′-end homology were identified for taxa with weaker conservation in the 5730 binding site, notably Giardia and other Metamonada. These include 5748–5647 (1,834 bp for *Giardia muris*) and 5753–5754 (1,343 bp). Slime moulds (e.g., *Fuligo septica* and *Polysphondylium pallidum*) have unusually expanded LSU regions (intron insertion) and therefore yield amplicons of 4–7 kb with most primer pairs. These taxa are best resolved with shorter-range primer pairs (e.g., 5390–5754) when expected. Detailed amplicon-size matrices for 17 ‘difficult’ and ‘key’ reference species across all 42 primer-pair combinations are provided in Supplementary Table S1.

**Table 2:** Predicted *in silico* amplicon sizes (bp) of the universal primer pair 5,753 and 5,730 across representative species spanning all major eukaryotic kingdoms.

Species	Kingdom/Supergroup	Phylum: Class: Order	NCBI accession	Amplicon (bp)
*Giardia muris*	Metamonada	Diplomonadida: Hexamitidae	X65063	1005
*Stephanocyclus meneghinianus*	SAR	Stramenopiles: Bacillariophyta	KY364696	1820
*Plasmodium berghei*	SAR	Alveolata: Apicomplexa: Aconoidasida	LK023127	2006
*Eimeria tenella*	SAR	Alveolata: Apicomplexa: Conoidasida	HG994970	2425
*Naegleria fowleri*	Percolozoa	Heterolobosea: Schizopyrenida	MZ430524	1475
*Cavenderia fasciculata*	Amoebozoa	Evosea: Eumycetozoa: Dictyostelia	DQ340389	2554
*Mucilago crustacea*	Amoebozoa	Myxogastria: Physarales	MZ313554	2428
*Fuligo septica*	Amoebozoa	Myxogastria: Physarales	AJ584697	4104
*Alternaria alternata*	Fungi	Ascomycota: Dothideomycetes	LC749799	1584
*Komagataella phaffii*	Fungi	Ascomycota: Saccharomycetes	CP014723	1075
*Saccharomyces cerevisiae*	Fungi	Ascomycota: Saccharomycetes	CP033498	1858
*Laetiporus sulphureus*	Fungi	Basidiomycota: Agaricomycetes	OV656687	1668
*Spirogloea muscicola*	Viridiplantae	Streptophyta: Zygnemophyceae	MN585752	1941
*Sphagnum subsecundum*	Plantae	Bryophyta: Sphagnopsida	MW528207	1865
*Marchantia polymorpha*	Plantae	Marchantiophyta: Marchantiopsida	AP019874	2739
*Perilla frutescens*	Plantae	Tracheophyta: Eudicots	KT220693	1735
*Aporrectodea icterica*	Animalia	Annelida: Clitellata: Opisthopora	OY744613	2442
*Danio rerio*	Animalia	Chordata: Actinopterygii: Cypriniformes	LR812082	2199
*Xenopus laevis*	Animalia	Chordata: Amphibia: Anura	X02995	2,460
*Drosophila melanogaster*	Animalia	Arthropoda: Insecta: Diptera	OY757196	2492
*Adineta vaga*	Animalia	Rotifera: Bdelloidea	GQ398061	2046
*Litomosoides sigmodontis*	Animalia	Nematoda: Chromadorea: Rhabditida	OY718804	2391
*Antedon bifida*	Animalia	Echinodermata: Crinoidea	OY727093	2065
*Perna viridis*	Animalia	Mollusca: Bivalvia: Mytilida	MK419106	2016
*Trichosurus vulpecula*	Animalia	Chordata: Mammalia: Marsupialia	ON496472	4143
*Homo sapiens*	Animalia	Chordata: Mammalia: Primates	NR_146144	4212
*Coturnix japonica*	Animalia	Chordata: Aves: Galliformes	OK523374	4401

Values are the distance between the outer ends of the two primer binding sites on the cited accession, that is, the length of the predicted PCR product, and not the length of the annotated rRNA gene cluster in that record. The product spans only the 3′ portion of 18S, ITS1, 5.8S, ITS2, and the 5′ portion of 28S, and is therefore necessarily much shorter than a full-operon annotation*.

^*^Two entries merit specific comment. Giardia muris (X65063) is annotated in GenBank with the historical designations “16S-like” and “23S-like” rRNA, which correspond to the 18S (SSU) and 28S (LSU) genes; Giardia also has an unusually compact rRNA gene unit with very short internal transcribed spacers, which is why the predicted product (1,005 bp) is much shorter than the approximately 4 kb annotated region. The same reasoning applies to Stephanocyclus meneghinianus (KY364696) and to the remaining entries: the tabulated value is the inter-primer distance, which depends on the position of the conserved priming sites within the operon and on the length of the intervening spacers, and is not comparable with the total length of the annotated feature. The complete 6 × 7 amplicon-size matrix, with the accessions used, is given in Supplementary Table S1.

### 3.2 PCR amplification of stool eukaryotic DNA

The 5753 and 5730 primer pair was applied to total stool DNA from 42 psoriasis patients and 24 healthy controls in single-tube long-range PCR. Distinct, sharp amplicons in the predicted 1.5–3.0 kb range were obtained from every sample, with no detectable bands in the corresponding extraction or PCR negative controls (Figure 1). Most samples yielded a dominant band at 1.6–1.9 kb consistent with fungal templates, with secondary bands at 1 kb (presumptive *Blastocystis*) and 2.5 kb (presumptive plant or arthropod DNA from diet) visible in a subset of patient lanes. Consistent with the design, no high-molecular-weight bands above 4 kb (the size predicted for the human host cassette) were observed, which is compatible with size-based under-representation of mammalian templates; the sequencing data presented below show that this suppression is substantial but incomplete.

**Figure 1:**
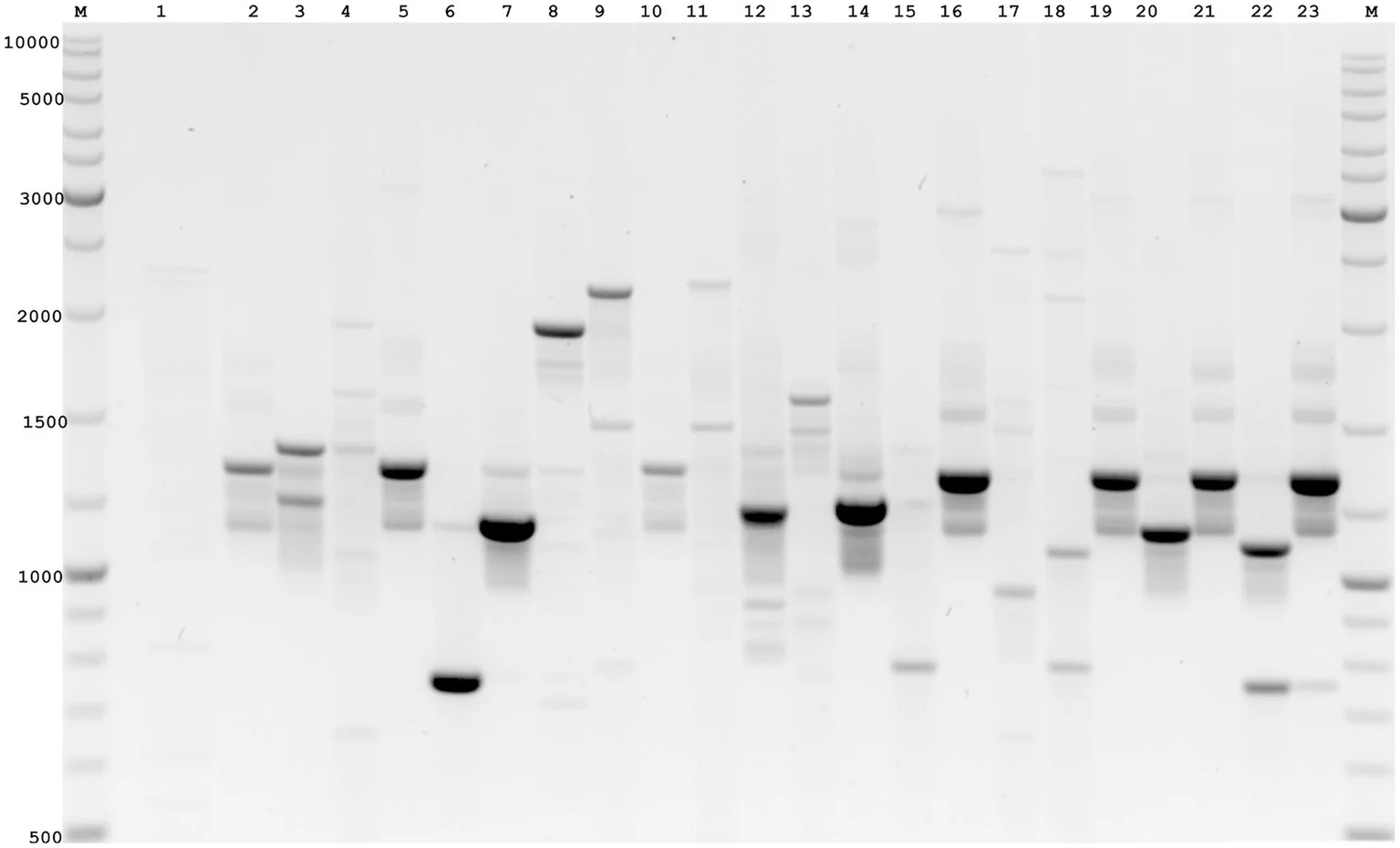
Universal long-range PCR amplification of eukaryotic rRNA genes from stool DNA. Agarose gel (1.5% w/v, 1 × TAE, 90 V, 3 h, ethidium bromide staining; ChemiDoc XRS+) of long-range PCR products generated with the universal eukaryotic primer pair (5753–5730). Lanes 1–23: Stool DNA from psoriasis patients (sample identifiers in Supplementary Table S2). Discrete amplicons in the predicted 1.5–3.0 kb range are visible in every patient lane; no high-molecular-weight bands above 4 kb (the size predicted for the human host rRNA gene cassette) were detected, consistent with size-based suppression of the host cassette by the assay. Size standard: GeneRuler DNA Ladder Mix (100–10,000 bp; Thermo Scientific SM0332).

### 3.3 Confirmation of amplicon identity by Sanger sequencing

To verify that the bands recovered from stool correspond to the intended eukaryotic cassette rather than to off-target products, individual bands were excised from agarose gels and sequenced by capillary electrophoresis. Sixteen amplicons generated with the 5728/5730 pair yielded high-quality consensus sequences of 966–1,115 bp (Supplementary Table S6; Supplementary Data S1). Every consensus contained the expected internal architecture of the cassette: the conserved 18S blocks recognised by primers 5751 and 5753 were present in all 16 sequences (0–2 mismatches), followed downstream by the 5763 and 5390 sites at the 18S-ITS1 boundary, confirming that the products span the intended 18S-ITS junction rather than an unrelated locus.

The sixteen amplicons resolved ten distinct taxa: Blastocystis hominis (three amplicons) and Blastocystis sp. (one); the fungi [*Candida*] *zeylanoides* (two), Debaryomycetaceae sp. (three), *Pichia kudriavzevii*, *Naganishia vishniacii*, *Trichosporon asahii*, Galactomyces geotrichum and *Torulaspora delbrueckii* (one each); and the intestinal amoeba *Endolimax nana* (one). Seven of these ten taxa were also recovered in the Nanopore dataset at genus level or better, including *Blastocystis hominis*, [*Candida*] *zeylanoides*, *Pichia kudriavzevii*, *Torulaspora delbrueckii* and *Debaryomyces* spp.; *Endolimax nana*, *Trichosporon asahii*, and *Galactomyces geotrichum* were not among the 687 entries retained from the barcoded libraries. Because the Sanger panel and the 32 barcoded libraries were prepared from different sets of stool samples, this is a taxon-level rather than a sample-level comparison, and the three non-overlapping taxa cannot be interpreted as false negatives of the Nanopore workflow. The recovery of *Endolimax nana*, an amoebozoan, alongside *Blastocystis* (*Stramenopiles*) and a range of Ascomycota and Basidiomycota nonetheless provides independent, sequence-level evidence that the cassette amplifies across distant eukaryotic lineages from a single stool reaction.

In the genus-selective configuration, primer 5780 paired with 5730 produced a single band of approximately 1.4 kb from *Blastocystis*-positive stool DNA. Consistent with this, the 5780 binding site was present internally, without mismatches, in four of the *Blastocystis*-assigned consensus sequences, at positions 300–310 of the 5728/5730 amplicon.

### 3.4 Multiplex long-range PCR validates broad cross-kingdom coverage with concurrent host-DNA suppression

To assess the practical utility of the universal primer panel under multiplex conditions, the format ultimately required for cost- and labour-efficient clinical deployment, we combined four 18S forward primers (5390, 5751, 5753, 5763) and two 28S reverse primers (5394, 5730) in a single PCR reaction and applied it to a phylogenetically diverse panel of template DNAs alongside total stool DNA from patients with plaque psoriasis (Figure 2). This built-in exclusion of host DNA is a key feature of the protocol when applied to stool, blood-contaminated, or biopsy specimens, where mammalian DNA would otherwise dominate sequencing depth and obscure the eukaryotic microbial signal. When applied to total stool DNA from four patients with plaque psoriasis (Figure 2), the multiplex panel produced highly reproducible amplicon profiles across samples. A dominant band at 1.4–1.5 kb was observed in every patient lane, consistent with the predicted size for *Saccharomycetaceae* (Table 2; *Saccharomyces cerevisiae* 1,593 bp; *Komagataella phaffii* 1,075 bp; *Candida* spp. estimated 1.3–1.5 kb), in agreement with the dominance of yeasts in the eukaryotic fraction of healthy and dysbiotic adult stool. A subset of samples additionally displayed a discrete higher-molecular-weight band at approximately 5 kb and a ladder of fainter products in the 1.2–1.7 kb range, indicative of co-amplification of multiple eukaryotic taxa, a pattern fully consistent with subsequent species-level resolution achieved by ONT sequencing. Together, these data indicate that the multiplex panel (i) retains the broad cross-kingdom coverage predicted *in silico*, (ii) delivers reproducible, sample-discriminating amplicon profiles from clinical stool DNA in a single reaction, and (iii) substantially reduces, without eliminating, amplification of host (mammalian and avian) DNA without requiring additional depletion steps three features that together render the workflow directly compatible with the rapid 96-sample ONT barcoding strategy described below.

**Figure 2:**
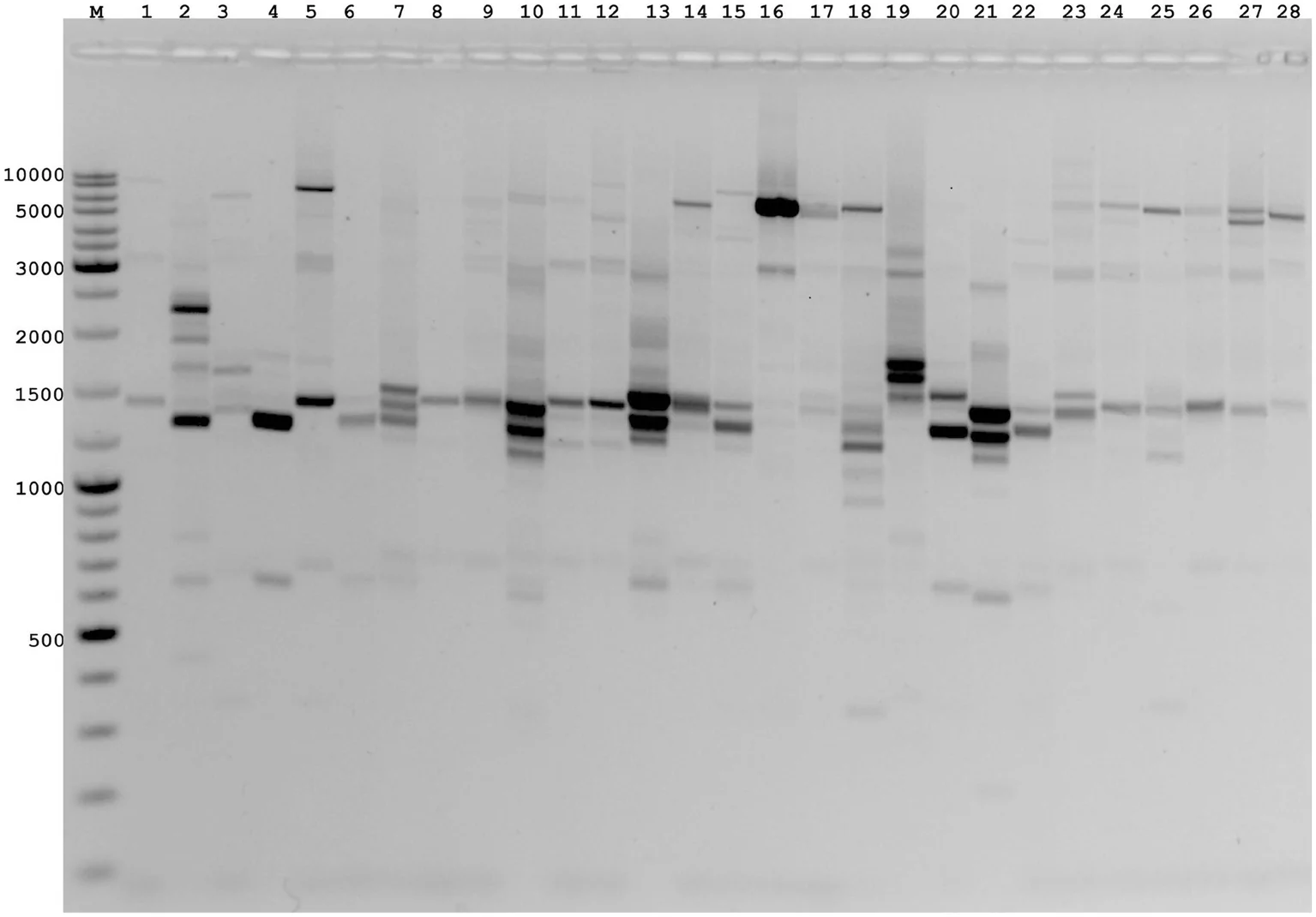
Single-tube multiplex long-range PCR amplification of the eukaryotic 18S-ITS1-5.8S-ITS2-28S rRNA gene cassette from human stool. A six-primer multiplex panel comprising four 18S forward primers (5390, 5751, 5753, 5763) and two 28S reverse primers (5394, 5730) was applied in a single reaction to DNA extracted from 28 individual stool samples (lanes 1–28). The assay amplifies a variable-length cassette of approximately 1.0–3.5 kb, with amplicon size reflecting the taxonomic composition of the eukaryotic fraction in each sample; multiple discrete bands within a single lane indicate co-amplification of phylogenetically distinct taxa (e.g., protists, fungi, and/or plant DNA) differing in ITS region length. Dominant bands at 1.2–1.5 kb correspond to the most prevalent eukaryotic constituents; higher-molecular-weight products (2–7 kb) reflect taxa with expanded ITS or variable regions. Lanes yielding faint or no product indicate low eukaryotic biomass in the corresponding sample. Lane M, GeneRuler DNA Ladder Mix (100–10,000 bp; Thermo Scientific SM0332); size markers (bp) are indicated on the left.

### 3.5 ONT amplicon sequencing performance

Barcoded libraries were sequenced on a single FLO-MIN106 (R9.4.1) flow cell on a GridION for 25 h, generating 4.07 Gb estimated and 2.9 Gb pass-quality bases (Q ≥ 9) from 4.83 million reads across all barcodes on the flow cell. The estimated read N50 was 1.07 kb. Live basecalling used a high-accuracy model at 450 bps (Dorado v7.2.13, MinKNOW v23.11.7). The 32 samples analysed in this study (barcodes 39–70) were processed with the EPI2ME wf-metagenomics v2.14.2 pipeline (Kraken 2.1.3 + Bracken v3.0.1) against the NCBI core_nt database (October 2025). After quality and length filtering (minimum read length 200 bp, minimum quality score 9), the pipeline retained a mean of 38,960 reads per sample (range 2,197-205,169). The mean unclassified fraction was 2.6 ± 2.1% (range 0.3–9.9%), with no samples exceeding 10%. After exclusion of non-eukaryotic off-target reads, Bracken eukaryotic classification yielded a mean of 30,778 ± 46,553 eukaryotic reads per sample (range 909–188,204; Supplementary Figures S1–S4). Negative controls (one extraction blank and one PCR no-template control) yielded 14–82 reads each (median 28), assigned exclusively to ubiquitous laboratory fungi (*Malassezia restricta*, *Cladosporium herbarum*, *Saccharomyces cerevisiae*); these values defined the contamination background for downstream filtering. Filtering of non-target assignments removed 108 bacterial, one archaeal, and six viral taxa, together with reads assigned to *Homo sapiens* and to *Macaca* spp., and five entries meeting the artefact criteria defined in Methods (*Photobacterium leiognathi*, *Poeciliopsis monacha*, *Mycoplasmopsis bovis*, *Arabidopsis thaliana* and synthetic constructs), leaving 687 eukaryotic species-level entries. Across the cohort, the eukaryotic fraction accounted for a mean of 30,778 of the 38,960 reads retained per sample, i.e., approximately four fifths of the classified material, so roughly one fifth of the sequenced reads were non-eukaryotic or host-derived. Host-derived and other chordate assignments were quantified systematically. After removal of *Homo sapiens* and *Macaca* spp., 29,801 of 984,884 classified eukaryotic reads (3.0%) remained assigned to Chordata; these were detected in 14 of 32 samples but exceeded 5% of the eukaryotic fraction in only three (BC43, 76.1%; BC58, 7.7%; BC51, 5.3%), and the cohort median was 0%. Eight entries corresponding to non-human primates (*Pan troglodytes*, *Pan paniscus*, *Gorilla gorilla*, *Pongo abelii*, *Nomascus leucogenys*, *Papio anubis*, *Callithrix jacchus*, *Saimiri boliviensis*) accounted for 552 reads in total, or 0.06% of the dataset, of which 532 came from BC43 alone; these are most plausibly residual human rRNA gene reads distributed across closely related hominid references rather than genuine primate DNA. BC43 was also the shallowest library in the cohort (909 classified eukaryotic reads), so its high proportional host content corresponds to fewer than 700 reads in absolute terms. Host suppression is therefore best described as a strong but incomplete, size-based depletion rather than as exclusion of host DNA. Representative per-sample taxonomic assignments for the 32 barcoded libraries are given in Table 3.

**Table 3:** Eukaryotic species identified by ONT 18S-28S amplicon sequencing in stool samples from Psoriasis patients and disease/healthy controls (representative subset; complete dataset in Supplementary Table S3).

Barcode	Sample ID	Group	Identified eukaryotic taxa
39	TP002	Psoriasis	*Saccharomyces cerevisiae*
40	TP003	Psoriasis	*Cladosporium herbarum, Saccharomyces cerevisiae*
41	TP005	Psoriasis	*Malassezia globosa, Candida parapsilosis*
42	TP008	Psoriasis	*Candida bombi, Saccharomyces cerevisiae*
43	TP009	Psoriasis	*Blastocystis hominis (ST3), Cladosporium herbarum*
44	TP010	Psoriasis	*Debaryomyces hansenii, Candida parapsilosis*
45	TP013	Psoriasis	*Candida parapsilosis, Saccharomyces cerevisiae*
46	TP014	Psoriasis	*Blastocystis hominis (ST3), Cladosporium herbarum*
47	TP016	Psoriasis	*Blastocystis hominis (ST1), Candida glaebosa*
48	TP018	Psoriasis	*Malassezia restricta, Saccharomyces cerevisiae*
49	TP021	Psoriasis	*Saccharomyces cerevisiae, Cladosporium herbarum*
50	TP022	Psoriasis	*Candida zeylanoides, Cladosporium herbarum*
51	TPK013	Healthy control	*Saccharomyces cerevisiae, Debaryomyces hansenii*
52	TPK015	Healthy control	*Blastocystis hominis (ST2), Saccharomyces cerevisiae*
53	TPK017	Healthy control	*Cladosporium herbarum, Saccharomyces cerevisiae*
54	TPK020	Healthy control	*Candida parapsilosis, Saccharomyces cerevisiae*
55	W12008	Disease control	*Blastocystis hominis (ST3)*
56	W12014	Disease control	*Blastocystis hominis (ST3)*
57	W12022	Disease control	*Candida zeylanoides*
58	TTK2110	Disease control	*Cladosporium herbarum, Blastocystis hominis (ST1)*

### 3.6 Eukaryotic taxonomic richness: 687 species-level entries from a single amplicon

After removal of non-eukaryotic reads and database cross-mapping artifacts, Bracken resolved 687 eukaryotic species-level taxa across the 32-sample cohort (Table 4). These comprised 297 Ascomycota, 102 Basidiomycota, 27 Mucoromycota, 63 Viridiplantae, 50 Arthropoda, 44 Chordata, 45 Protista (including Blastocystis), 14 SAR (Stramenopiles and Alveolata), 6 Nematoda, and other eukaryotic lineages. Mean per-sample eukaryotic richness was 63.4 ± 55.5 (median 37.5; range 4–197), Shannon H′ was 1.96 ± 0.77 (range 0.31–3.70), and Simpson 1-D was 0.66 ± 0.22 (range 0.10–0.93; Figure 3; Table 4).

**Figure 3:**
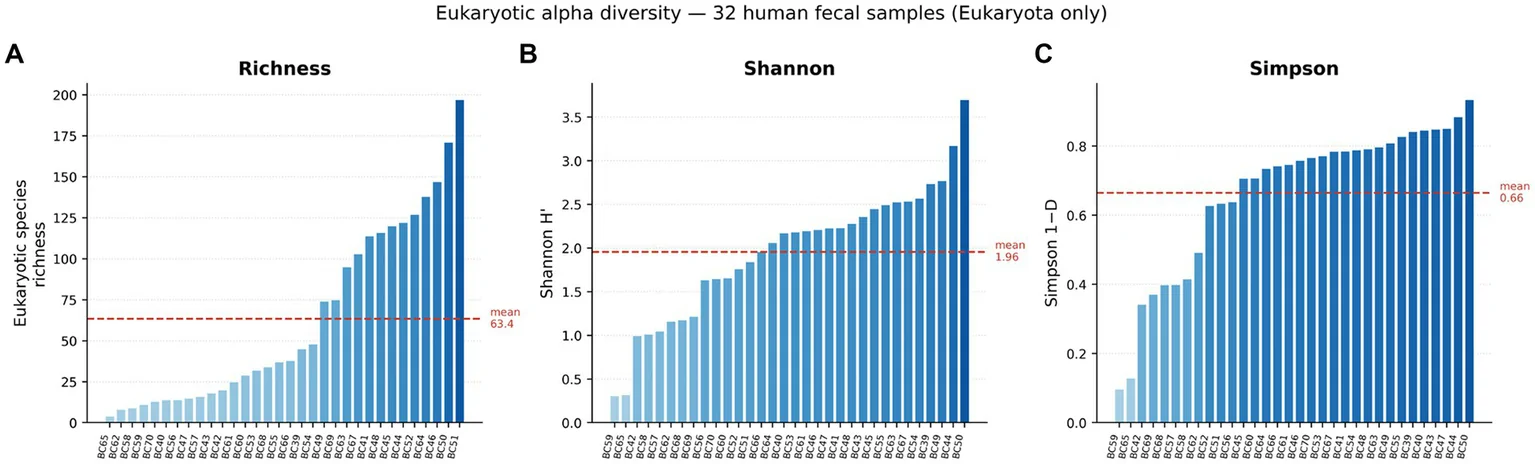
Eukaryotic alpha diversity across 32 human stool samples (barcodes BC39-BC70; Eukaryota only). **(A)** Observed species richness (mean 63.4; range 4–197). **(B)** Shannon entropy index H′ (mean 1.96; range 0.31–3.70). **(C)** Simpson diversity index 1-*D* (mean 0.66; range 0.10–0.93). Bars are sorted in ascending order within each metric; colour intensity reflects relative rank. The dashed red line indicates the cohort mean. Samples BC65, BC59, and BC58 showed the lowest diversity, corresponding to single-taxon-dominated eukaryotic profiles (*Anethum graveolens*, *Starmerella apicola*, and *Brassica rapa*/*Allium sativum*, respectively). BC51 (highest richness: 197) and BC50 (highest Shannon and Simpson) were *Blastocystis*-dominated high-depth samples. All metrics were computed from Bracken-corrected eukaryotic abundance tables after removal of database cross-mapping artefacts.

**Table 4:** Alpha diversity summary and sequencing quality metrics across 32 gut metagenomic samples.

Metric	Mean ± SD	Median (IQR)	Range
Species richness	63.41 ± 55.50	37.50 (15.75–114.50)	4–197
Shannon diversity (H′)	1.96 ± 0.77	2.18 (1.53–2.46)	0.31–3.70
Simpson diversity (1-D)	0.66 ± 0.22	0.75 (0.59–0.80)	0.10–0.93
Classified eukaryotic reads	30,778 ± 46,553	9,155 (3,335–30,275)	909–188,204

This table summarises the alpha diversity indices and sequencing depth characteristics of the gut eukaryome across all analysed samples. Species richness ranged from 4 to 197 detected taxa per sample, with a mean richness of 63.4 ± 55.5 and a median of 37.5, indicating substantial inter-individual heterogeneity in taxonomic composition. Shannon diversity averaged 1.96 ± 0.77 (range: 0.31–3.70), reflecting moderate diversity with strong variation in taxon distribution among individuals. Simpson diversity (1-D) showed a mean value of 0.66 ± 0.22 (range: 0.10–0.93), suggesting that while some samples were dominated by a few taxa, others exhibited relatively even community structures. The number of classified eukaryotic reads per sample ranged from 909 to 188,204, with a mean of 30,778 reads, confirming sufficient sequencing depth for comparative ecological analysis while also highlighting marked differences in eukaryotic signal intensity across samples. Overall, these results demonstrate that the human gut eukaryome is highly heterogeneous, with both low-diversity and highly enriched community profiles observed across the cohort.

### 3.7 Eukaryotic community composition

Fungi (Ascomycota + Basidiomycota) dominated the eukaryotic fraction in 22/32 samples, while Protista (predominantly *Blastocystis*) dominated in 10/32 samples (Figure 4). Viridiplantae (dietary plant DNA) accounted for >50% of eukaryotic reads in 5 samples (BC42, BC57, BC58, BC65, BC68), reflecting dietary contributions from *Allium sativum* (garlic), *Anethum graveolens* (dill), and *Brassica rapa*. The genera represented by the largest numbers of species-level database entries were *Candida* (51 entries, 37 of them named binomials), *Cladosporium* (37), *Blastocystis* (27 entries, 12 of them subtype-labelled), *Malassezia* (20), *Aspergillus* (14), *Debaryomyces* (11), *Saccharomyces* (9), *Starmerella* (9), *Penicillium* (8), and *Kazachstania* (8) (Table 5; Supplementary Figures S1–S4).

**Figure 4:**
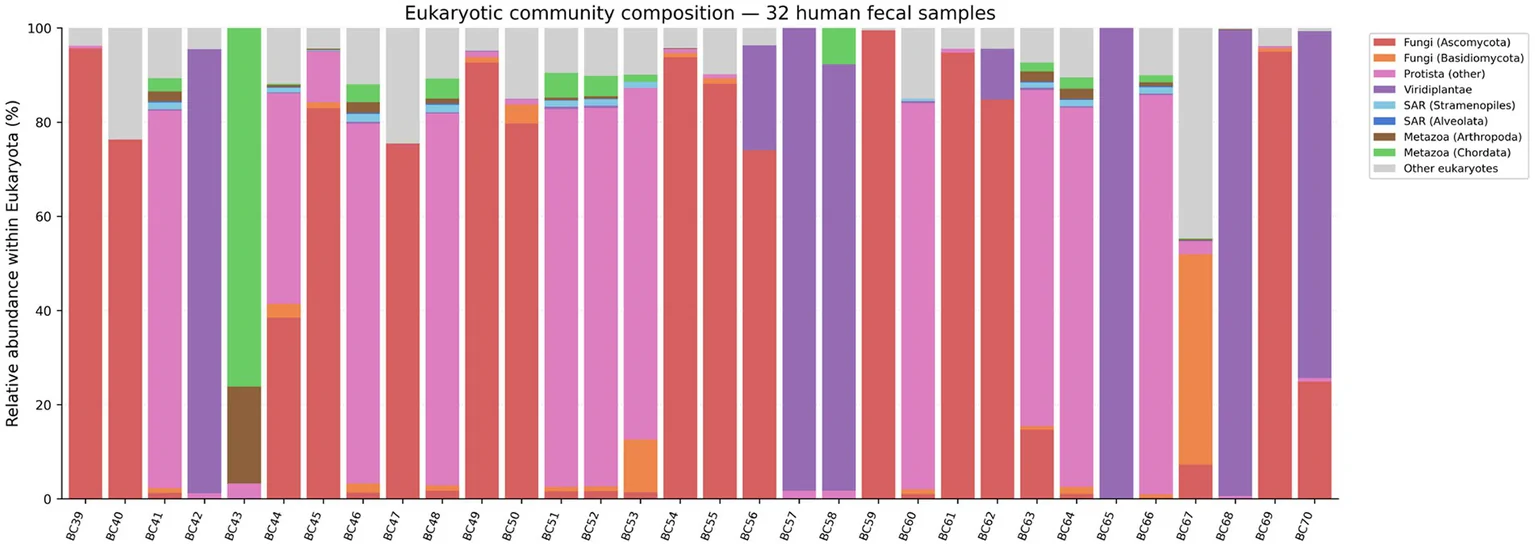
Eukaryotic community composition across 32 human stool samples. Stacked bars show relative abundance (%) within the eukaryotic fraction, grouped by major lineage: fungi (Ascomycota, red), fungi (Basidiomycota, orange), Protista (other, including *Blastocystis*, pink), Viridiplantae (purple), SAR Stramenopiles (light blue), SAR Alveolata (dark blue), Metazoa Arthropoda (brown), Metazoa Chordata (green), and other eukaryotes (grey). Fungi (Ascomycota) dominated in the majority of samples; Protista dominated in 10 samples (*Blastocystis* carriers: BC41, BC44, BC46, BC48, BC51, BC52, BC60, BC63, BC64, BC66). Viridiplantae accounted for >80% of eukaryotic reads in BC42 (*Anethum graveolens*), BC57, BC58 (*Allium sativum*), BC65, and BC68 (*Brassica rapa*/*oleracea*). BC43 showed anomalous Metazoa (Chordata) dominance (76%), consistent with residual host rRNA gene amplification. Classification: Kraken 2.1.3/Bracken v3.0.1; NCBI core_nt (October 2025); non-eukaryotic reads excluded.

**Table 5:** Prevalence and relative abundance of the most frequently detected eukaryotic taxa across 32 human stool samples.

Species	Prevalence (%)	Mean rel. Abund. (%)	Max rel. Abund. (%)	Eukaryotic group
Uncultured fungus	87.5	7.55	43.55	Fungi (other)
Uncultured eukaryote	78.1	1.85	10.00	Protista (other)
*Saccharomyces cerevisiae*	75.0	5.06	79.07	Fungi (Ascomycota)
*Brassica rapa*	59.4	1.50	28.13	Viridiplantae
Haptophyta environmental samples	50.0	0.05	0.23	Haptophyta
*Kazachstania servazzii*	40.6	0.39	1.96	Fungi (Ascomycota)
*Blastocystis hominis*	37.5	7.54	58.51	Protista (other)
*Claroideoglomus etunicatum*	37.5	0.38	2.85	Fungi (Mucoromycota)
*Penicillium soppii*	37.5	0.04	0.17	Fungi (Ascomycota)
Uncultured Ascomycota	37.5	0.06	0.69	Fungi (Ascomycota)
*Tricholoma matsutake*	34.4	0.11	0.50	Fungi (Basidiomycota)
*Cymatosiraceae* sp.	34.4	0.16	1.11	SAR (Stramenopiles)
*Schizophyllum commune*	34.4	0.06	0.37	Fungi (Basidiomycota)
*Candida albicans*	34.4	1.55	21.67	Fungi (Ascomycota)
*Candida tropicalis*	34.4	0.80	9.24	Fungi (Ascomycota)
*Humulus lupulus*	31.3	0.10	1.06	Viridiplantae
*Candida parapsilosis*	31.3	5.20	70.16	Fungi (Ascomycota)
*Geotrichum candidum*	31.3	0.10	0.82	Fungi (Ascomycota)
*Homarus americanus*	31.3	0.03	0.17	Metazoa (Arthropoda)
*Leptosphaeria biglobosa*	31.3	0.09	1.14	Fungi (Ascomycota)
Uncultured Glomeromycotina	31.3	0.11	0.88	Fungi (Mucoromycota)
*Meyerozyma guilliermondii*	28.1	0.44	4.57	Fungi (Ascomycota)
*Neoechinorhynchus agilis*	28.1	0.02	0.13	Metazoa (other)
*Chaetoceros tenuissimus*	28.1	0.24	1.16	SAR (Stramenopiles)
*Wickerhamomyces anomalus*	28.1	0.25	3.92	Fungi (Ascomycota)

For each taxon, the table reports prevalence, mean, and maximum relative abundance within the eukaryotic fraction (%), and eukaryotic group classification. Taxa are sorted by descending prevalence; the table shows all species detected in ≥25% of samples. Uncultured fungus was the sole core eukaryotic taxon (≥80% prevalence; mean 7.6%). *Saccharomyces cerevisiae* (75.0%) and *Brassica rapa* (59.4%) reflect ubiquitous dietary contributions. *B. hominis* ((37.5%) is the most clinically relevant protist detected, with *B. hominis* reaching up to 58.5% of eukaryotic reads in individual carriers. *Candida albicans*, *C. tropicalis* (34.4% each), and *C. parapsilosis* (31.2%) are pathobionts associated with Th17-mediated immune activation in psoriasis. *Claroideoglomus etunicatum* (Glomeromycota; 37.5%) is an arbuscular mycorrhizal fungus of plant origin. *Tricholoma matsutake* (Basidiomycota; 34.4%) and *Schizophyllum commune* (34.4%) represent environmental and dietary fungal exposures.

### 3.8 Core eukaryome structure and sparsity

The eukaryotic abundance matrix was highly sparse (90.8% zero entries; Table 6), indicating a strongly individualised gut eukaryome. Of the 687 detected taxa, 313 (45.6%) were singletons present in only one sample, and 503 (73.2%) occurred in fewer than 10% of samples. Only five taxa met the threshold for common prevalence (≥50%): uncultured fungus (87.5%), uncultured eukaryote (78.1%), *Saccharomyces cerevisiae* (75.0%), *Brassica rapa* (59.4%), and *Haptophyta* environmental samples (50.0%) (Supplementary Figure S3). A single taxon (uncultured fungus) qualified as a core eukaryotic taxon (≥80% prevalence). These prevalence-based descriptors apply to the sequencing depths achieved here and are re-examined under depth normalisation below. These findings indicate that the human gut eukaryome, as sampled by this assay, is characterised by high inter-individual variability and low prevalence overlap, with a small universal core (Table 6), in contrast to the conserved bacterial core microbiome; the depth-dependence of the last of these observations is examined below.

**Table 6:** Core eukaryome structure, prevalence distribution, and sparsity characteristics across 32 gut metagenomic samples.

Metric	Value
Total samples	32
Total detected taxa	687
Total abundance matrix cells	21984
Non-zero cells	2029
Zero cells	19955
Matrix sparsity	90.8%
Matrix occupancy	9.2%
Singleton taxa (present in 1 sample)	313
Rare taxa (<10% prevalence)	503
Moderately prevalent taxa (10–49%)	179
Common taxa (≥50%)	5
Core taxa (≥80%)	1
Median taxa per sample	37.5
Mean taxa per sample ± SD	63.4 ± 55.5

This table summarises the structural properties of the gut eukaryome, including prevalence distribution, abundance matrix sparsity, and the extent of the stable core community across all samples. A total of 687 eukaryotic taxa were detected across 32 samples, generating an abundance matrix of 21,984 taxa-sample observations. Of these, only 2,029 cells (9.2%) contained non-zero values, while 19,955 cells (90.8%) were zeros, indicating a highly sparse and individualised eukaryotic community structure. More than 45% of all detected taxa were singletons (313 taxa), present in only one sample, while 503 taxa occurred in less than 10% of samples, demonstrating strong zero inflation and low taxonomic consistency across individuals. Only five taxa were classified as common (≥50% prevalence), and only one taxon met the definition of a core taxon (≥80% prevalence), supporting the concept of a minimal stable gut eukaryotic core. The median number of taxa detected per sample was 37.5, while the mean richness was substantially higher (63.4 ± 55.5), reflecting strong inter-individual variability and the presence of highly enriched samples. Overall, these findings indicate that the human gut eukaryome is characterised by high sparsity, low prevalence overlap, and substantial personalisation rather than a conserved universal core community.

### 3.9 Blastocystis prevalence and subtype diversity

Using a conservative threshold of ≥50 Bracken-assigned reads, *Blastocystis* was detected in 12/32 subjects (37.5%). *Blastocystis* sp. (aggregate) reached a mean of 12.1% relative abundance within the eukaryotic fraction (max 44.9%), while *B. hominis* was confirmed at the species level in all 12 carriers (mean 7.5%, max 58.5%). Twenty-seven Blastocystis-assigned entries were retained, comprising three named species, one unresolved “*Blastocystis* sp.” entry, 12 subtype-labelled entries (ST1-ST4, ST7, ST10-ST15, ST24) and 11 named isolate entries, including SP-2014, R44, RN94-9, and C14 (Table 6). These are database entries recovered by k-mer classification, not subtype calls verified by the standard barcoding-region approach (Stensvold et al., 2020). At the Bracken detection floor of ≥ 10 reads per taxon, ST14 was the most frequently detected subtype-labelled entry (37.5% of subjects), followed by ST3 (34.4%), ST4 (31.2%), and ST2 (31.2%); applying the more conservative ≥ 50-read threshold used above, the corresponding values were 34.4, 31.2, 15.6, and 21.9%, respectively. Subtype-level entries cannot be distinguished by a 250–500 bp 18S V4 fragment, but no such comparison was performed on these samples, and the assignments reported here require independent confirmation before they can be treated as subtype determinations.

### 3.10 Candida species: psoriasis-relevant pathobionts

With a ≥ 50-read threshold, *Candida* spp. were detected in 13/32 subjects (40.6%), encompassing 51 species-level entries assigned to the genus, 37 of which correspond to named binomials. Three species of established clinical relevance to psoriasis were detected at high prevalence: *C. albicans* (34.4%; mean 0.8%, max 15.1%), *C. tropicalis* (34.4%; max 5.5%), and *C. parapsilosis* (31.2%; max 45.0%). Additional pathobiont species included [*Candida*] *glabrata* (31.2%), *C. dubliniensis* (9.4%; max 15.6%), and [*C.*] *palmioleophila* (25.0%). Multiple entries assigned to *Candida* co-occurred within individual stool samples, an observation compatible with the concept of a polymicrobial *Candida* gut reservoir (Table 5); the present design does not test that concept, and no association with disease status was evaluated.

### 3.11 *Malassezia*: enteric colonization beyond the skin

With a ≥ 10-read threshold, the assay detected *Malassezia* in 4/32 subjects (12.5%), with 20 species-level entries assigned to the genus, 15 of which corresponded to named species: *M. restricta* (9.4%; up to 12.1% relative abundance), *M. globosa* (9.4%; max 3.0%), *M. sympodialis* (6.2%), *M. furfur* (6.2%), *M. pachydermatis* (6.2%), and 15 additional species at 3.1% prevalence.

### 3.12 Dietary and environmental eukaryotes

*Saccharomyces cerevisiae* was the most prevalent fungal species (75.0%; mean 5.1%, max 79.1%), reflecting both endogenous gut colonisation and dietary intake. *Debaryomyces hansenii* (59.4%; max 48.6%) and *Starmerella apicola* (3.1%; 95.0% in BC59, dominating that sample) were additional prominent Ascomycota. Environmental fungi included *Cladosporium* spp. (37 species) and *Penicillium* spp. (8 species), consistent with airborne/dietary exposure. Dietary plant DNA was recovered at the species level: *Brassica rapa* (59.4%), *Allium sativum* (garlic; 6.2%; up to 76.9%), *Anethum graveolens* (dill; 9.4%; up to 93.2%), and *Brassica oleracea* (28.1%; max 29.5%). Recovery of plant DNA at species-level resolution directly from stool is a potentially useful by-product of the assay; no dietary records were collected in this study, so these detections could not be verified against reported food intake (see Discussion).

### 3.13 Beta diversity

Bray-Curtis PCoA based exclusively on the eukaryotic fraction revealed substantial inter-sample variation with no discrete clustering (Supplementary Figure S3), consistent with the high sparsity (90.8%) and individualisation of the gut eukaryome of the participants. Samples with the lowest Shannon diversity (BC59, BC65, and BC58) were positioned at the periphery of the ordination space, reflecting their atypical composition dominated by single taxa (BC59:95.0% *Starmerella apicola*; BC65:100% Viridiplantae).

### 3.14 Sensitivity of diversity and core estimates to sequencing depth

Sequencing depth varied more than 200-fold across the cohort (909–188,204 classified eukaryotic reads; median 9,155; coefficient of variation 151%), and observed richness was strongly correlated with depth (Spearman ρ = 0.89, *p* = 1.8 × 10^−11^). Diversity and core analyses were therefore repeated under depth normalisation (Supplementary Table S5; Supplementary Figure S6). Rarefaction of all 32 samples to the depth of the shallowest library (909 reads) reduced mean richness from 63.4 ± 55.5 to 40.7 ± 28.5 entries, while mean Shannon diversity was essentially unchanged (1.96 ± 0.77 versus 1.93 ± 0.75), indicating that the depth effect acted mainly on the detection of rare entries rather than on dominant community structure. The rank correlation between richness and library size decreased after rarefaction but remained significant (ρ = 0.80, *p* = 4 × 10^−8^), which suggests that samples yielding more eukaryotic reads also tend to harbour genuinely more complex eukaryotic communities rather than being merely better sampled. Matrix sparsity was robust to normalisation: 90.8% in the observed matrix, 91.8% after rarefaction to 909 reads, 88.6% at 3,000 reads (24 samples), 86.8% at 5,000 reads (21 samples), and 81.9% at 10,000 reads (15 samples). Excluding rather than rarefying shallow libraries gave the same picture (88.0% sparsity for the 24 samples with at least 3,000 reads; 86.4% for the 21 samples with at least 5,000 reads). The size of the core, by contrast, is clearly depth-dependent: the number of entries reaching 50% prevalence rose from 3 (rarefied to 909 reads) to 9, 18, and 37 at 3,000, 5,000, and 10,000 reads, and the number reaching 80% prevalence rose from 1 to 3, 3, and 10, respectively. The identity of the most prevalent entries was nevertheless stable across all normalisations, with uncultured fungus, uncultured eukaryote, and *Saccharomyces cerevisiae* ranking first at every depth, followed by *Brassica rapa*, *Haptophyta* environmental samples, *Blastocystis* spp., and *Claroideoglomus etunicatum*. Genus-level prevalence estimates were also robust to the choice of threshold. *Blastocystis* prevalence was 37.5% whether defined by at least 50 assigned reads or by at least 0.1% or 1% of the eukaryotic fraction; *Candida* prevalence was 34.4% at both the 50-read and the 0.1% thresholds and 31.2% at the 1% threshold; *Malassezia*, which occurs at much lower abundance, fell from 12.5% (10-read threshold) to 9.4% (0.1%) and 6.2% (1%), so its prevalence estimates are threshold-dependent and are reported here with the threshold stated. Taken together, these analyses indicate that the sparsity of the gut eukaryome and the small number of highly prevalent entries are not artefacts of shallow sequencing, but that the absolute size of the core is a function of sequencing effort and should not be interpreted as a fixed biological quantity.

## 4 Discussion

We developed and applied a broad-range long-read amplicon sequencing assay for the human gut eukaryome, combining a rationally designed pan-eukaryotic 18S–28S primer set with the rapid-barcoding workflow of Oxford Nanopore Technologies and a Dorado-minimap2-Kraken2 bioinformatic pipeline. The assay simultaneously profiles fungi, protists, helminth-relevant clades, and dietary plant material from a single PCR product, generated of the order of 10,000–100,000 classified eukaryotic reads per sample in a 25-h run on a single flow cell, in a barcoding format that accommodates up to 96 multiplexed samples, and reaches species- (and frequently subtype-) level taxonomic resolution. To our knowledge, this is the first description of a broad-range eukaryotic 18S-28S amplicon protocol explicitly engineered to (i) operate as two-step long-range PCR with no degenerate bases, (ii) under-represent host (mammalian) DNA by amplicon-size selection, and (iii) be paired with Kraken2 classification against an explicitly eukaryote-enriched k-mer database. The workflow retained 687 species-level database entries from 32 samples using a single 18S-28S primer pair classified against the NCBI core_nt (October 2025) database with Kraken 2.1.3 and Bracken v3.0.1. By restricting the analysis exclusively to the eukaryotic fraction, the intended target of the 18S-28S amplicon, and applying stringent database artefact filtering, we provide a characterisation of the gut eukaryome from which off-target bacterial assignments have been removed, although, as discussed below, removing such assignments does not establish that the corresponding molecules were never amplified.

Conventional eukaryote metabarcoding targets the V4 or V9 regions of the 18S rRNA gene (250–500 bp) or the fungal ITS1/ITS2, yielding amplicons that frequently cannot discriminate *Candida* species, *Malassezia* species, or *Blastocystis* subtypes (Pawlowski et al., 2012; Tedersoo et al., 2015). The 1.0–3.4 kb cassette amplified by our 5753/5730 pair encompasses the entire 3′-half of 18S (including V8 and V9), the complete 5.8S gene, both ITS1 and ITS2, and the 5′-third of 28S (D1-D3 expansion segments), providing substantially more sequence context per read than a 250–500 bp V4 or V9 fragment. Because the cassette is recovered as a single contiguous molecule, chimeras between separately amplified marker regions cannot arise; PCR-mediated chimeras between co-amplified templates within the same reaction remain possible, however, and were not screened for in this study, so the absence of chimeras cannot be asserted (Edgar et al., 2011; Edgar, 2016). The retention of 51 species-level entries assigned to *Candida*, 20 *Malassezia*, 37 *Cladosporium*, and 14 *Aspergillus* (37, 15, 23 and 12 of them named binomials, respectively) exceeds what short-read protocols typically report. These are, however, counts of database entries retained after filtering rather than verified species detections, and no full-ITS or full-28S amplicon was generated from the same samples for comparison. The 18S-ITS-28S cassette is therefore best regarded as an optimised compromise for stool broad cross-kingdom coverage, an amplicon length (1.0–3.5 kb) compatible with fragmented, low-input DNA, and workable long-range PCR efficiency rather than as a universally superior marker. Longer 28S-spanning amplicons carry more phylogenetic information but increase the risk of PCR failure and chimera formation when the template is fragmented, which is the usual situation in stool.

A defining feature of our dataset is its extreme sparsity: 90.8% of the taxon-sample matrix cells are zero, 45.6% of detected taxa are singletons, and only one taxon (uncultured fungus) exceeds 80% prevalence (Table 6). This contrasts with the conserved bacterial core microbiome and is compatible with a gut eukaryome shaped primarily by stochastic colonisation, individual dietary habits, and environmental exposure rather than host selection for a stable symbiotic community. The depth-sensitivity analyses reported above show that this sparsity is not an artefact of shallow sequencing, but also that the number of entries qualifying as core rises with sequencing effort, from one at approximately 900 reads to 10 at 10,000 reads; the “minimal core” is therefore a statement about detection at a given depth rather than a fixed property of the community. The low core size has practical implications: eukaryome studies require substantially larger sample sizes to identify disease-associated taxa than equivalent bacteriome studies.

Three findings are of translational relevance. First, the high prevalence and species diversity of gut *Candida* (34.4% for both *C. albicans* and *C. tropicalis*; 51 entries in total) shows that the assay recovers the taxa central to the “fungal hypothesis” of psoriasis, in which candidalysin activates epidermal IL-36γ/IL-17 circuits cross-reactive with psoriatic keratinocyte antigens. This hypothesis was not tested here: no differential-abundance or case–control analysis was performed, and these detections must not be read as evidence of an association with psoriasis in this cohort. Second, the recovery of 20 *Malassezia* species from stool, including *M. restricta* at up to 12.1%, adds to evidence that enteric *Malassezia* has been implicated in CARD9-dependent gut-skin axis inflammation (Limon et al., 2019; Spatz and Richard, 2020). Whether enteric *Malassezia* is associated with psoriasis in this population is a hypothesis for adequately powered case–control studies rather than a finding of the present work. Third, the concurrent recovery of 12 subtype-labelled *Blastocystis* entries from a single amplicon offers a potential epidemiological platform for investigating *Blastocystis*-psoriasis associations that is not available from conventional methods. The overall *Blastocystis* prevalence of 37.5% is within the range reported for adult populations in Central Asia (Stensvold et al., 2020; Piperni et al., 2024), and the most frequently detected subtype-labelled entries were ST14 (37.5% of subjects) and ST3 (34.4%), the latter being the subtype most consistently linked to gastrointestinal symptoms and bacteriome perturbations. Because subtype assignment here rests on k-mer classification against subtype-labelled database entries, and because R9.4.1 reads retain indel errors in homopolymeric tracts, these assignments are provisional and require confirmation by barcoding-region subtyping (Stensvold et al., 2020) before epidemiological use.

The species-level recovery of *Allium sativum* (garlic; up to 76.9%), *Anethum graveolens* (dill; up to 93.2%), *Brassica rapa* (59.4% prevalence), and *B. oleracea* from stool demonstrates that the 18S-28S amplicon recovers plant-derived DNA from stool alongside microbial sequences. No dietary records were collected, so these detections cannot be linked to documented food intake, and the data do not establish that the assay reconstructs dietary exposure; validation against food-frequency questionnaires, weighed dietary records, or controlled feeding studies would be required before use in nutritional epidemiology. Plant DNA is also recoverable from stool using short-read ITS1 protocols (Defazio et al., 2026); therefore, the distinctive feature of the present workflow is that plant, fungal, and protist signals are obtained from the same amplicon rather than the detection of dietary DNA.

For studies in which dietary plant DNA is the primary target, the panel includes a Viridiplantae-selective reverse primer (5765; Table 1), which would allow plant sequences to be amplified selectively rather than recovered as a by-product of pan-eukaryotic amplification.

Eight species-level entries were removed as probable classification artefacts: three *Macaca* species (primate rRNA gene cross-mapping with host), *Photobacterium leiognathi* (100% prevalence indicating systematic misclassification), and *Poeciliopsis monacha* (freshwater fish). Additionally, all non-eukaryotic reads (108 bacterial, 6 viral, 1 archaeal) were excluded as off-target amplification products. Researchers employing Kraken2 with the core_nt database should implement similar post-hoc filtering and report minimum read-count thresholds for prevalence claims. The recovery of prokaryotic, viral, and host sequences from an amplicon library is expected rather than anomalous and follows directly from the library chemistry. Rapid transposase-based barcoding (SQK-RBK110-96) is sequence-agnostic: the transposase tagments and tags every double-stranded DNA molecule present in the reaction, not only the intended PCR product. Post-PCR clean-up does not remove this background and in fact concentrates it, because a 0.6 × AMPure XP ratio is a large-fragment selection: unconsumed template DNA carried over from the 25 ng of total stool DNA used in each reaction is retained preferentially, while primers and short by-products are discarded. Since stool metagenomic DNA is overwhelmingly bacterial, a carryover mass fraction far too small to produce a visible band or smear on an agarose gel is sufficient to generate a measurable read fraction, and the same mechanism accounts for the human, primate, and viral assignments. Two observations are consistent with this interpretation: no discrete non-target bands were detected on the gels (Figures 1, 2), so the non-eukaryotic reads are unlikely to represent abundant off-target PCR products; and the negative controls carried through library preparation yielded only 14–82 reads each, assigned exclusively to ubiquitous laboratory fungi, so the non-eukaryotic signal derives from the nucleic acids of the samples themselves rather than from reagent contamination. Neither observation is decisive, however: an agarose gel cannot exclude a diffuse, low-mass background of high-molecular-weight DNA. Two further mechanisms may therefore contribute. The first is genuine off-target priming on the prokaryotic 16S-23S rRNA gene region, which carries the same universally conserved motifs and is far more abundant than eukaryotic rRNA genes in stool, where bacterial genomes outnumber eukaryotic ones by orders of magnitude. The second is k-mer cross-mapping, in which reads originating from conserved eukaryotic rRNA gene regions are assigned to a prokaryotic reference. Consistent with a real off-target contribution, the eukaryotic fraction accounted for only approximately four fifths of the reads retained per sample. Distinguishing the two mechanisms requires amplicon-level verification Sanger or targeted long-read sequencing of gel-purified off-target bands, or realignment of the discarded reads against 16S and 23S references and is a priority for the next iteration of the protocol. Until such verification is available, the assay is described here as strongly enriching for, rather than exclusively amplifying, eukaryotic templates, and the removed entries are described as probable rather than demonstrated artefacts. Therefore, carryover during library preparation is the most parsimonious explanation for the non-eukaryotic fraction, but it is not a demonstrated one, and genuine off-target priming and k-mer cross-mapping may contribute in parallel. These three mechanisms make different and readily testable predictions. Reads derived from the carryover template should map across the donor genomes in proportion to genome size and should follow the fragment-length distribution of the input DNA. True off-target PCR products should instead pile up on the 16S-23S rRNA gene region, since that is where the conserved motifs recognised by the primers are located, and should show the discrete length distribution characteristic of an amplicon. Cross-mapped reads should align preferentially to conserved rRNA gene blocks while retaining eukaryotic sequences at the variable positions. Realigning the discarded reads against the corresponding reference genomes separates these possibilities directly, and we regard this as a necessary step before non-target assignments in long-read amplicon data are interpreted. Practical mitigation is equally straightforward: excision of the target band from the gel or a tighter size-selection strategy before library preparation would reduce carryover at the source. Until such verification is available, researchers employing Kraken2 with the core_nt database should apply post-hoc filtering of the kind described here, report minimum read-count thresholds for prevalence claims and treat removed entries as probable rather than demonstrated artefacts. Similar to all amplicon-based approaches, this assay is semi-quantitative. Nuclear rRNA gene copy numbers vary by more than two orders of magnitude across eukaryotes, from a few dozen copies in some protists to hundreds in fungi and thousands in plants and animals, and they vary even between strains of the same fungal species (Prokopowich et al., 2003; Torres-Machorro et al., 2010; Lofgren et al., 2019). Amplification efficiency also depends on amplicon length, and the cassette targeted here spans 1.0–3.5 kb; therefore, shorter templates are expected to be overrepresented in a mixed reaction. Relative read abundances therefore reflect the combined effect of biomass, copy number, and amplification efficiency and should be read as ordinal indicators of community structure rather than as estimates of biomass. The data are also compositional; therefore, any future differential-abundance analysis will require compositionally aware methods (Gloor et al., 2017). The completeness of the reference database is a first-order limitation for eukaryotic metabarcoding. Even in core_nt, many gut protists and non-model fungi are represented only by partial 18S sequences or unidentified environmental entries: 43 of the 687 retained entries were “uncultured” or “environmental sample” records, they carried 12.7% of all classified eukaryotic reads, and one of them (uncultured fungus) was the most prevalent entry in the cohort, present in 87.5% of samples although accounting for only 8.5% of reads. This is a property of the reference space rather than of the amplicon: a read spanning 18S-ITS-28S carries ample information for species-level assignment, but an assignment can only be as precise as the closest available reference. Curated, taxonomically resolved full-length eukaryotic rRNA gene references, for example, an extension of PR2 and UNITE (Guillou et al., 2013; Nilsson et al., 2019) to complete 18S-ITS-28S cassettes, would convert a substantial part of the current “uncultured” signal into named taxa. ONT R9.4.1 chemistry retains a residual indel error profile in homopolymer regions even after super-accuracy basecalling; migration to R10.4.1/V14 chemistry is recommended for clinical-grade deployment. Residual indel errors in homopolymeric regions particularly affect discrimination between near-identical references; therefore, both the *Blastocystis* subtype-level entries and the assignment of closely related Candida species should be regarded as provisional pending confirmation on R10.4.1 data or by an orthogonal method. The observed read N50 of 1.07 kb shorter than the predicted 1.5–3.0 kb amplicon range indicates that a substantial fraction of sequenced molecules were fragmented, likely reflecting the degraded nature of stool-extracted DNA and the shearing that occurs during bead-beating lysis. The 1.0–3.5 kb amplicon window excludes organisms with very large rRNA gene operons (some slime moulds and ciliates). Some taxa detected at very low abundance (<0.01%) may represent residual database cross-mapping rather than true biological signals. The high inter-sample variance in read depth (CV > 150%; range 909–188,204 reads) indicates that diversity estimates in low-depth samples are subject to rarefaction bias. One sample (BC43) retained 76.1% Metazoa (Chordata) reads within the eukaryotic fraction, reflecting residual host rRNA gene amplification despite the amplicon-size discrimination design; this sample should be interpreted with caution, although in absolute terms it contributed fewer than 700 chordate reads, and cohort-wide chordate assignments amounted to 3.0% of classified eukaryotic reads. The most important limitation of this study is the absence of a defined mock community. The primer set was evaluated *in silico* against curated kingdom-representative references and experimentally on stool and on a panel of template DNAs; however, amplification bias, detection limits, reproducibility, quantitative distortion, and taxonomic accuracy cannot be quantified without mixtures of known composition; for this reason, the assay is described here as a broad-range, pan-eukaryotic profiling approach rather than as a universally validated method. Second, no head-to-head benchmark against conventional short-read 18S V4/V9 or ITS1/ITS2 protocols was performed on the same samples; therefore, statements about relative resolution rest on the sequence content of the amplicons rather than on empirical comparison. Third, species- and subtype-level assignments in the Nanopore dataset were made by k-mer classification alone. Sanger sequencing of 16 individual gel-purified amplicons confirmed the identity of the cassette and of 10 taxa spanning Stramenopiles, Amoebozoa, Ascomycota and Basidiomycota, but it was performed on a separate set of stool samples and therefore validates the assay rather than the individual assignments reported for the 32 barcoded libraries; systematic confirmation of those assignments by alignment against curated references, phylogenetic placement, or targeted PCR remains outstanding. Fourth, no chimera detection was applied, and the chimera rate of this long-range multi-template PCR is unknown. Finally, the cohort sequenced here was small and not balanced by design; no case–control statistical comparison was performed, and the psoriasis samples served as a clinical application example rather than as evidence of disease association. Priorities for further development are as follows: benchmarking against a defined mock eukaryotic community and, on the same samples, against conventional short-read 18S V4/V9 and ITS1/ITS2 protocols; migration to R10.4.1 chemistry with consensus polishing; independent confirmation of key taxonomic assignments by alignment to curated references, phylogenetic placement, or targeted PCR; extension to at least 200 patients and matched controls per dermatosis with longitudinal sampling and adequately powered, compositionally aware differential-abundance analysis; integration of 18S-28S and 16S amplicons on the same flow cell for cross-kingdom co-occurrence networks; and standardisation of sequencing depth, with rarefaction or coverage-based diversity estimation applied as implemented here.

## 5 Conclusion

We present a proof-of-concept, end-to-end protocol for broad-range long-read amplicon sequencing of the human gut eukaryome, comprising (i) a panel of rationally designed pan-eukaryotic 18S-28S rRNA gene primers free of degenerate bases and engineered to suppress host-amplicon co-amplification by amplicon-size discrimination; (ii) a single-tube long-range PCR compatible with rapid-barcode ONT library preparation; (iii) a high-throughput sequencing workflow on a single FLO-MIN106 flow cell; and (iv) a reproducible bioinformatic pipeline (wf-metagenomics v2.14.2; Kraken 2.1.3/Bracken v3.0.1) paired with the NCBI core_nt database (October 2025), the most current publicly available eukaryote-enriched reference for k-mer-based classification. Application to 32 stool samples from a clinical cohort of plaque-psoriasis patients and matched controls retained 687 eukaryotic species-level database entries after artefact filtering, including 51 species-level entries assigned to *Candida* (37 named binomials), 27 entries assigned to *Blastocystis* (12 subtype-labelled entries), and 20 entries assigned to *Malassezia* (15 named species) recovered from a single PCR product. The gut eukaryome exhibited high sparsity (90.8%) and marked individualisation; only one entry reached 80% prevalence at the depths achieved here, a number that rises with sequencing effort. Psoriasis-relevant pathobionts *C. albicans* (34.4%), *C. tropicalis* (34.4%), and *M. restricta* (9.4%; up to 12.1%) were simultaneously detected alongside dietary plant DNA. The assay therefore enables the broad simultaneous recovery of eukaryotic sequences from human stool and provides more sequence context for taxonomic classification than short-read markers. Further validation using defined mock communities, independent confirmation of key taxonomic assignments, standardised sequencing depth, and larger, balanced clinical cohorts will be required before it can be regarded as a fully validated platform for clinical or population-scale eukaryome profiling.

## Data Availability

Raw Oxford Nanopore sequencing reads (FASTQ) generated from the 32 stool samples have been deposited in the NCBI Sequence Read Archive (SRA) under BioProject accession PRJNA1462757 (https://www.ncbi.nlm.nih.gov/bioproject/PRJNA1462757). The curated 18S-28S eukaryotic reference database, the universal primer set, and processing scripts used in this study are openly available on Zenodo under DOI: 10.5281/zenodo.10702287 and 10.5281/zenodo.10574384. The code implementing the sequencing-depth sensitivity, core-robustness and host-read analyses reported here (eukaryomeQC v1.0.0), together with the command lines, parameter files and software versions used for read classification, is available at: https://github.com/rkalendar/eukaryomeQC. In line with FAIR data practice, an anonymised metadata table giving group assignment (psoriasis or control), sex, and age band for every sequenced sample is provided as Supplementary Table S2; under the Law of the Republic of Kazakhstan No. 94-V of 21 May 2013 “On Personal Data and Their Protection” (as amended), depersonalised data from which it is impossible to determine their belonging to a data subject (Article 1(7)) fall outside the protection obligations of owners and operators, which run only until the data are destroyed or depersonalised (Article 22(2)), and depersonalisation is in any case mandatory where personal data are processed for scientific research (Article 17(1)). Publication of the depersonalised table is therefore permitted under Kazakhstan law and is covered by the written informed consent obtained from all participants and by the ethics approvals cited above. Variables that could permit re-identification in a cohort of this size, such as exact sampling dates, individual PASI trajectories, or free-text comorbidity records, remain available from the corresponding author on reasonable request and subject to approval by the institutional ethics committee.
