# Supplementary material for: Targeted long-read eukaryotic profiling of human stool by broad-range 18S–28S rRNA gene amplicon sequencing on Oxford Nanopore Technologies: a proof-of-concept study
Source: Front Microbiol. 2026 Sep 30;17:1904425. doi: 10.3389/fmicb.2026.1904425 (PMC13664300; doi:10.3389/fmicb.2026.1904425)
Supplement: Supplementary file 1 [file Supplementary_file_1.DOCX]

**Supplemental Figures**

**Figure S1. Top-25 eukaryotic taxa by mean relative abundance across 32 human stool samples (log₁₀-scaled heatmap).** Colour intensity represents log₁₀ relative abundance within the eukaryotic fraction (white = absent or <0.01%; dark blue = ≥100%). *Blastocystis* sp. and *B. hominis* are the highest-abundance taxa, reaching >40% in multiple carriers. *Anethum graveolens* (dill) dominates BC42, BC68, and BC67, reflecting dietary intake. *Saccharomyces cerevisiae* shows a near-ubiquitous low-abundance presence, with a single high-abundance sample (BC69: 79.1%). *Allium sativum* (garlic) reaches 76.9% in BC56-BC57. *Starmerella apicola* dominates BC59 (95%). Dietary plant species (*Brassica rapa*, *B. oleracea*), environmental fungi (*Cladosporium* sp.), and multiple *Candida* and *Blastocystis* subtypes are visible as sample-specific signatures. Classification: Bracken v3.0.1; NCBI core_nt (October 2025).

**Figure S2. Bray-Curtis principal coordinates analysis (PCoA) of 32 human fecal samples based on eukaryotic species composition only.** Each point represents one sample; colour indicates Shannon diversity index H′ (light = low, dark = high). No discrete clustering by clinical group was observed, consistent with the high sparsity (90.8%) and individualisation of the human gut eukaryome. Notable outliers: BC59 (far left; lowest diversity; 95% *Starmerella apicola*), BC43 (upper right; 76% Chordata reads), and BC65/BC42 (upper cluster; Viridiplantae-dominated). Samples with high *Blastocystis* abundance (BC41, BC46, BC48, BC51, BC52, BC63, BC64, BC66) cluster in the lower-central quadrant. Ordination performed by non-metric multidimensional scaling (scikit-learn MDS, random_state = 42) on pairwise Bray-Curtis distances computed from relative abundance profiles of 687 eukaryotic species.

**Figure S3.** **Top-20 eukaryotic taxa by prevalence across 32 human stool samples.** Horizontal bars indicate the percentage of samples in which each taxon was detected (≥1 Bracken-assigned read). Values to the right of bars indicate mean relative abundance within the eukaryotic fraction. Bar colour denotes eukaryotic group. Only one taxon (uncultured fungus) reached ≥80% prevalence, reflecting the extreme individualisation of the gut eukaryome. *Blastocystis* sp. and *B. hominis* (37.5%) and *Candida albicans* (34.4%) represent clinically relevant taxa detected at the applied ≥50-read threshold. *Brassica rapa* (59.4%) and *Haptophyta* environmental samples (50%) reflect dietary and environmental inputs. *Claroideoglomus etunicatum* (Mucoromycota, Glomeromycetes) is an arbuscular mycorrhizal fungus detected in 37.5% of samples. Classification: Bracken v3.0.1; NCBI core_nt (October 2025).

 **Figure S4. Eukaryotic classified read counts per sample. Bars are sorted by descending read count.** BC51 (188,204 reads) and BC64 (133,772) yielded the highest eukaryotic depth; BC47 (1,052) and BC43 (909) the lowest. Dashed red line indicates the cohort mean (30,778 reads). The high coefficient of variation (CV >150%) reflects heterogeneity in stool DNA quality, PCR yield, and eukaryotic biomass across samples. Counts represent Bracken-corrected species-level assignments to eukaryotic taxa only, after removal of non-eukaryotic off-target reads and database cross-mapping artefacts.

**Figure S5. Recruitment, sampling and analysis flow.** Participants were recruited in Phase I of a cross-sectional clinical study (2017–2018) comprising 91 plaque-psoriasis patients and 92 matched healthy controls. In Phase II, 20 psoriasis patients and 20 controls (n = 40) were selected at random for stool eukaryome profiling. DNA extracts were accepted for amplification if they showed a discrete high-molecular-weight band on the integrity gel, an A260/A280 ratio of 1.8–2.0 and an A260/A230 ratio of approximately 2.0. Accepted extracts were amplified with the 5753/5730 long-range primer pair and with the multiplex panel, barcoded using the Rapid Barcoding Kit 96, and sequenced on a single FLO-MIN106 (R9.4.1) flow cell. Eight libraries failed the pre-defined quality criteria and were excluded before analysis, leaving 32 samples (barcodes BC39–BC70) for taxonomic profiling with Kraken2/Bracken against NCBI core_nt (October 2025), which retained 687 eukaryotic species-level entries after removal of non-eukaryotic, host-derived and artefactual assignments. Group composition of the analysed set is given in Supplementary Table S2.

**Figure S6. Sensitivity of diversity and core estimates to sequencing depth.** (A) Rarefaction curves for all 32 samples; each line represents one library, plotted on a log₁₀-scaled read axis. The dashed vertical line marks the depth of the shallowest library (909 classified eukaryotic reads), the common depth used for whole-cohort normalisation. Curves for the deepest libraries have not reached saturation, indicating that further sequencing would recover additional eukaryotic entries. (B) Observed entries per sample plotted against library size before (red) and after (blue) rarefaction to 909 reads. The rank correlation between richness and library size falls from ρ = 0.89 to ρ = 0.80 but remains significant, indicating that the richness gradient is only partly explained by sampling effort. (C) Matrix sparsity (purple line, left axis) across rarefaction depths of 909, 3,000, 5,000 and 10,000 reads; the grey dashed line marks the sparsity of the unnormalised matrix (90.8%). Bars (right axis) give the number of entries reaching 50% (blue) and 80% (red) prevalence at each depth. Sparsity is stable across normalisations, whereas the number of entries qualifying as core rises from 1 to 10, showing that core size is a function of sequencing effort rather than a fixed property of the community. Samples below each target depth are omitted at that depth, so the four points are based on 32, 24, 21 and 15 samples respectively. (D) Percentage of the eukaryotic fraction assigned to Chordata (grey) and to non-human primate references (red) in each sample, sorted in descending order. Residual host signal exceeds 5% in only three samples; BC43, the shallowest library in the cohort, is dominated by primate-assigned reads, which correspond to fewer than 700 reads in absolute terms. Analyses performed with eukaryomeQC v1.0.0 (50 subsamplings per depth, seed 20260818); numerical values are given in Supplementary Table S5.
