# Supplementary material for: Targeted long-read eukaryotic profiling of human stool by broad-range 18S–28S rRNA gene amplicon sequencing on Oxford Nanopore Technologies: a proof-of-concept study
Source: Front Microbiol. 2026 Sep 30;17:1904425. doi: 10.3389/fmicb.2026.1904425 (PMC13664300; doi:10.3389/fmicb.2026.1904425)
Supplement: Supplementary file 2 [file Data_Sheet_1.ZIP › LaboratoryNotes.pdf]

# Supplementary Data S1

## Laboratory notebook records for primer development and confirmatory Sanger sequencing

**Supporting:** Kalendar R, Daniyarov A, Kairov U, Kozhakhmetov S, Yerezhepov D, Yermekbayeva B, Sarbassov D, Kushugulova A. Targeted long-read eukaryotic profiling of human stool by broad-range 18S-28S rRNA gene amplicon sequencing on Oxford Nanopore Technologies: a proof-of-concept study. Manuscript 1904425.

### Purpose of this document

This file reproduces the primary laboratory record of the wet-lab work that underlies two claims in the manuscript: that the broad-range 18S-28S primer pairs amplify a single dominant product from human faecal DNA, and that the identity of that product was verified by capillary (Sanger) sequencing of individual gel-purified bands. It is provided so that the amplification conditions, template panels, gel images and band-to-sample assignments behind those claims can be inspected directly rather than taken on trust, in line with current expectations for data and metadata availability.

The record covers nineteen amplification experiments performed between January and April 2024. It documents the screening of candidate forward and reverse primers, the selection of the pairs reported in Table 1 of the manuscript, the amplification of the specific bands that were excised and sequenced, and the development of the two lineage-selective configurations: the Blastocystis-selective pairing (primer 5780 with the broad-range 28S reverse primer 5730) and the Viridiplantae-selective pairing (the plant-specific 28S reverse primer 5765 with the broad-range 18S forward primer 5751).

### What is included

- LaboratoryNotes.docx (this file) - dated experiment entries, each with the primer combination, the template panel with lane assignments, the reaction composition, the thermal cycling programme, and the agarose gel image.
- PCR_amplicons.fasta - consensus sequences of the sixteen individually sequenced amplicons (966-1,115 bp).
- SangerSequences/ - the seventeen raw electropherograms (.ab1) from which those consensus sequences were called, run on 3 April 2024.

### How to read an entry

Each entry begins with a heading of the form YYYYMMDD, followed by the assay and the primer combination in parentheses, and the template material. The line beginning with "M" lists the gel lanes in loading order, "M" denoting the size marker (GeneRuler 1 kb DNA Ladder). The table headed "DNA:" maps lane numbers to template identifiers. The paragraph headed "PCR" gives the composition of the master mix and the volume dispensed per tube, followed by the cycling programme. Entries appear in reverse chronological order, with the exception of the first entry.

Template identifiers follow three conventions used in the laboratory: alphanumeric clinical codes (for example TP002, W12008, TП014) denote stool DNA extracts from the study cohort; codes of the form 1C, 24C-1, 43c-2 denote individual purified extracts from the working panel; and TPC-prefixed codes denote the pilot extraction series. Sample identifiers in this record are internal laboratory codes and are not linked to any participant-identifying information.

### Relationship to the sequenced amplicons

Sequence identifiers in PCR_amplicons.fasta have the form index(lane:sample)_assignment, for example 3(9:TPC7)_Blastocystis sp. The lane numbers correspond to the gel of 26 March 2024, on which all sixteen sequenced templates appear; the dedicated re-amplification of the selected subset for sequencing is recorded under 27 March 2024. All sixteen amplicons were generated with the 5728/5730 primer pair. Per-amplicon identifications, consensus lengths and correspondence with the Nanopore dataset are given in Supplementary Table S6.

## Summary of experiments

| Date | Primer pair(s) | Templates | Purpose |
|---|---|---|---|
| 24 Jan 2024 | 5765 / 5751 | 13 plant DNAs (Gagea, Tulipa), pooled mix, 18 environmental extracts | First test of the Viridiplantae-selective pairing; strong uniform product from purified plant DNA, little or no product from environmental DNA |
| 25 Jan 2024 | 5765 / 5751 vs 5756 / 5751 | 12 plant DNAs per pair | Head-to-head comparison of the plant-selective and the broad-range reverse primer on identical templates |
| 03 Feb 2024* | 5728 / 5730 | 44 clinical stool DNA extracts | First application of the broad-range pair to the clinical panel; repeated with five additional cycles |
| 26 Mar 2024 | 5728 / 5730 | 43 extracts (TPC and C series) | Screening run; defines the lane numbering used in the sequence identifiers |
| 27 Mar 2024 | 5728 / 5730 | 19 selected extracts | Dedicated re-amplification of selected templates for band excision and Sanger sequencing |
| 28 Mar 2024 | 5728 / 5730 | 23 extracts (C series) | Control run on an independent template set |
| 29 Mar 2024 | 5753 / 5756 | 23 extracts (C series) | Same templates with an alternative primer pair |
| 01 Apr 2024 | 5751 with 5756, 5754, 5759, 5758, 5760 | 8 extracts per pair | Side-by-side comparison of five reverse primers against a common forward primer |
| 02 Apr 2024 | 5753 / 5756 | 44 clinical stool DNA extracts | Alternative pair applied to the full clinical panel |
| 03 Apr 2024 | 5751 with 5756, 5754, 5759, 5758, 5760 | 8 extracts per pair | Repeat of the reverse-primer comparison |
| 03-04 Apr 2024 | 5751 / 5758 | 16 extracts | Test of the longest amplicon configuration, with extended elongation time |
| 09 Apr 2024 | 5728 / 5730 | 19 extracts, new DNA | Broad-range pair on a freshly extracted panel, single-temperature elongation |
| 10 Apr 2024 | 5751 / 5756 | 46 extracts, new DNA | Alternative pair on the same fresh panel |
| 12 Apr 2024 | 5728, 5779, 5780, 5781, each with 5730 | 1 template, 4 reactions | Screen of four candidate forward primers for a Blastocystis-selective assay |
| 18 Apr 2024 | 5780 / 5730 | 82 extracts | Selected Blastocystis-selective pair applied at panel scale |
| 19 Apr 2024 | 5780 / 5730 | 87 extracts | Continuation across the extended panel |
| 20 Apr 2024 | 5780 / 5730 | 41 extracts plus repeats | Final application of the Blastocystis-selective pair |

* recorded in the notebook as "202400203"; the intended date is 3 February 2024.

## Note on scope

This is a working laboratory record, reproduced without retrospective editing of the original entries. The descriptive paragraph placed under each heading has been added for this submission and states the purpose and design of the experiment; it does not add measurements that were not recorded at the time. Gel images are reproduced as captured, and band sizes quoted in the manuscript were read against the marker lanes shown here.

**20240412 18S-28S (18S rRNA only *Blastocystis* sp.) primers test**

*Screen of four candidate forward primers - 5728, 5779, 5780 and 5781 - each paired with the broad-range eukaryotic 28S reverse primer 5730, with the aim of identifying a forward primer that anchors in an 18S block conserved within Blastocystis but not across eukaryotes. A common master mix containing template, buffer and the reverse primer was dispensed into four tubes, and the individual forward primers were added separately, so that the four lanes differ only in the forward primer. The 5780/5730 combination was carried forward and is the pair used in all subsequent entries and reported in the manuscript.*

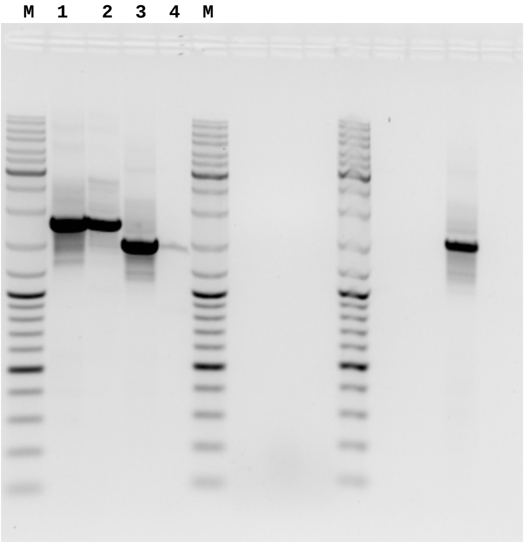


**Primer mix:**
**1** – 5728-5730
**2** – 5779-5730
**3** – 5780-5730
**4** – 5781-5730

**PCR**
150 µl reaction mix contains 2.5 µl DNA, 94 µl MilliQ, 30 µl 5x Phusion HF Buffer, 3 µl dNTP (10 mM), 1.5 µl Thermo Scientific™ Phusion HS II Hot Start DNA Polymerase (2 U/µL), 6 µl 5 µM primer 5730 (final **200 nM**).
Mix briefly and transfer 30 µl mix to PCR tube and add 1.2 µl 5 µM primer.

PCR amplification was carried out under the following conditions:
initial denaturation step at 98°C for **90 sec**, followed by **34x** amplifications at 98°C for **5 s**, **68°C for 30s**, **72°C for 20s**, followed by a final extension of 72°C for 1 min.

**20240420 18S-28S (18S rRNA only *Blastocystis* sp.) primers test (5780-5730) DNA human**

*Application of the selected Blastocystis-selective pair 5780/5730 to a panel of 41 human stool DNA extracts, with repeat reactions from the runs of 18 and 19 April loaded alongside (lanes 85-87 and 80-82) for direct comparison on the same gel. The expected product is approximately 1.4 kb.*

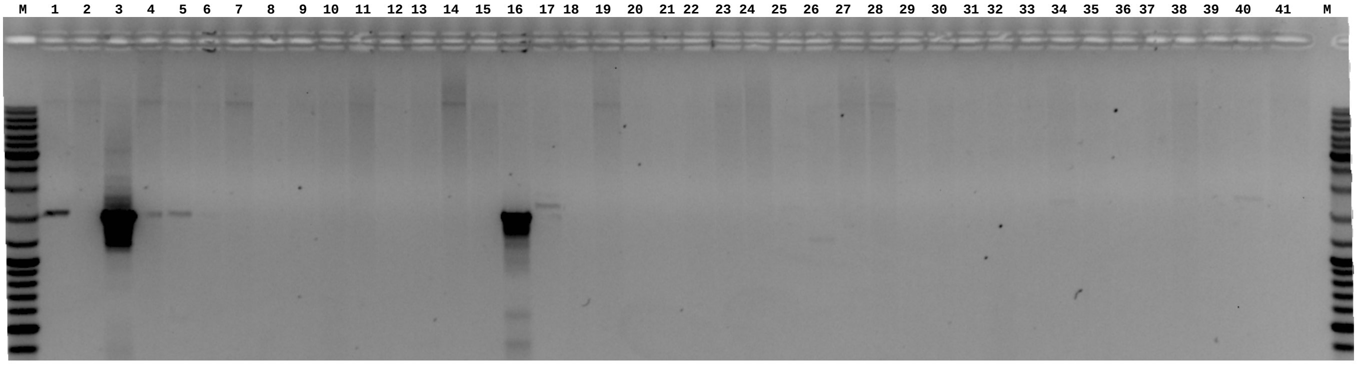


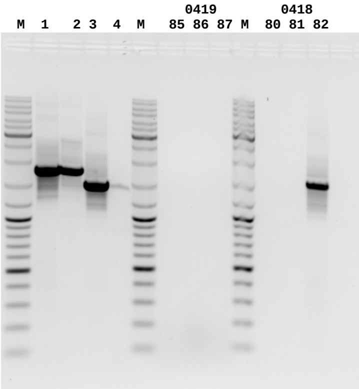


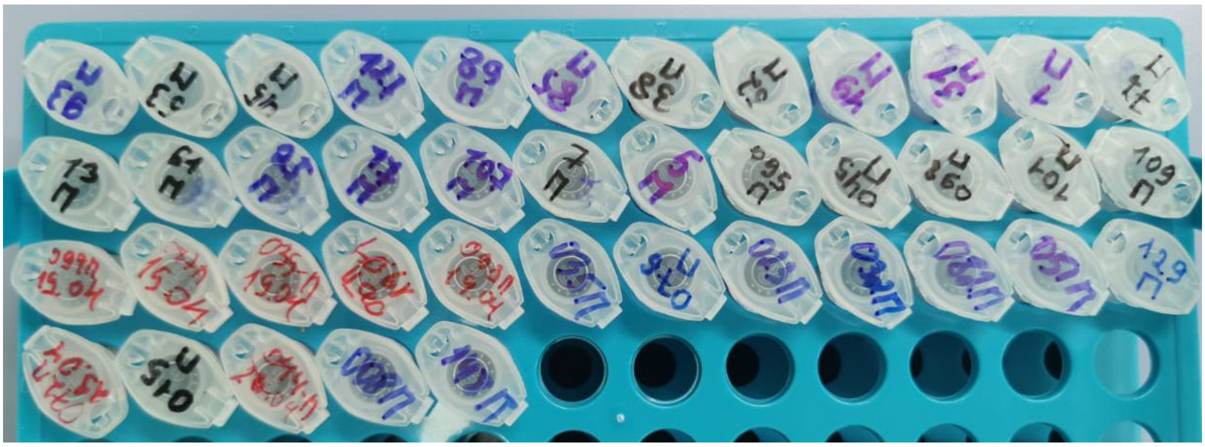

**PCR**

900 µl reaction mix contains 580 µl MilliQ, 180 µl 5x Phusion HF Buffer, 18 µl dNTP (10 mM), 9 µl Thermo Scientific™ Phusion HS II Hot Start DNA Polymerase (2 U/µL), each 36 µl 5 µM primers (final **200 nM**).
Mix briefly and transfer 19 µl mix to PCR tube and add 1 µl DNA (30-60 ng/µl).

PCR amplification was carried out under the following conditions:
initial denaturation step at 98°C for **90 sec**, followed by **34x** amplifications at 98°C for **5 s**, **68°C for 30s**, **72°C for 20s**, followed by a final extension of 72°C for 1 min.

**20240419 18S-28S (18S rRNA only *Blastocystis* sp.) primers test (5780-5730) DNA human**

*Continuation of the 5780/5730 screen across the extended panel of 87 stool DNA extracts, loaded on two gels. Reaction volume was scaled to 1,850 µl of master mix to keep the composition identical across the whole panel.*

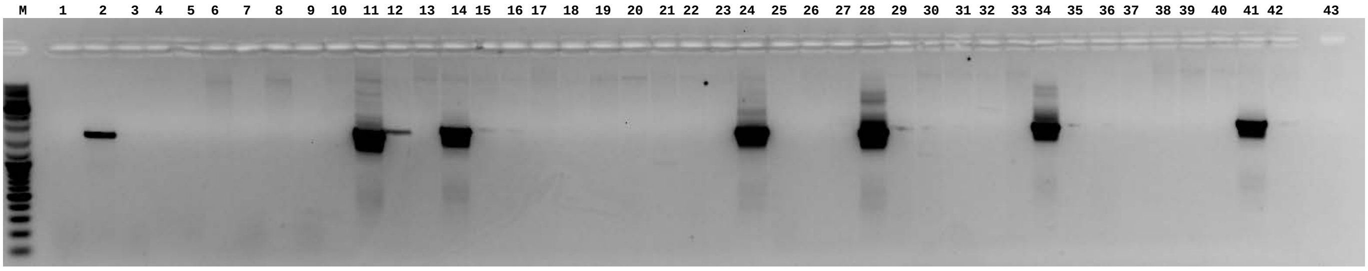


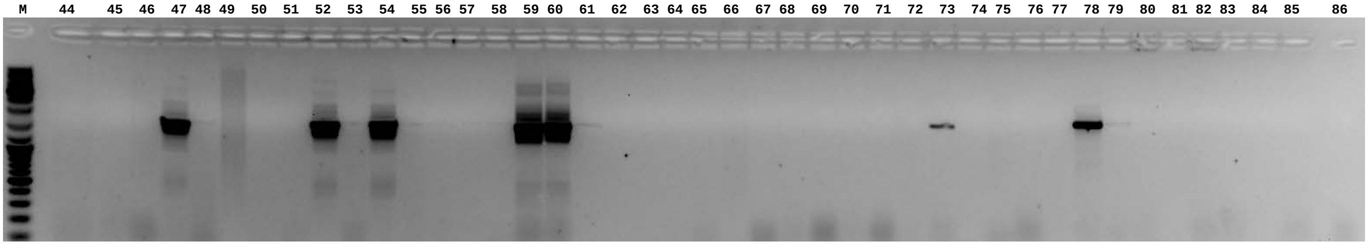


**DNA: 1-87 ()**

**PCR**

1850 µl reaction mix contains 1185 µl MilliQ, 370 µl 5x Phusion HF Buffer, 37 µl dNTP (10 mM), 18 µl Thermo Scientific™ Phusion HS II Hot Start DNA Polymerase (2 U/µL), each 74 µl 5 µM primers (final **200 nM**).

Mix briefly and transfer 19 µl mix to PCR tube and add 1 µl DNA (30-60 ng/µl).

PCR amplification was carried out under the following conditions:

initial denaturation step at 98°C for **90 sec**, followed by **34x** amplifications at 98°C for **5 s**, **68°C for 30s**, **72°C for 20s**, followed by a final extension of 72°C for 1 min.

**20240418 18S-28S (18S rRNA only *Blastocystis* sp.) primers test (5780-5730) DNA human**

*Application of the 5780/5730 pair to 82 stool DNA extracts (identifiers MT01 to X1 and 40-1-12-1), loaded on two gels. Conditions are identical to the runs of 19 and 20 April, so the three gels together constitute a single panel-scale evaluation of the Blastocystis-selective assay.*

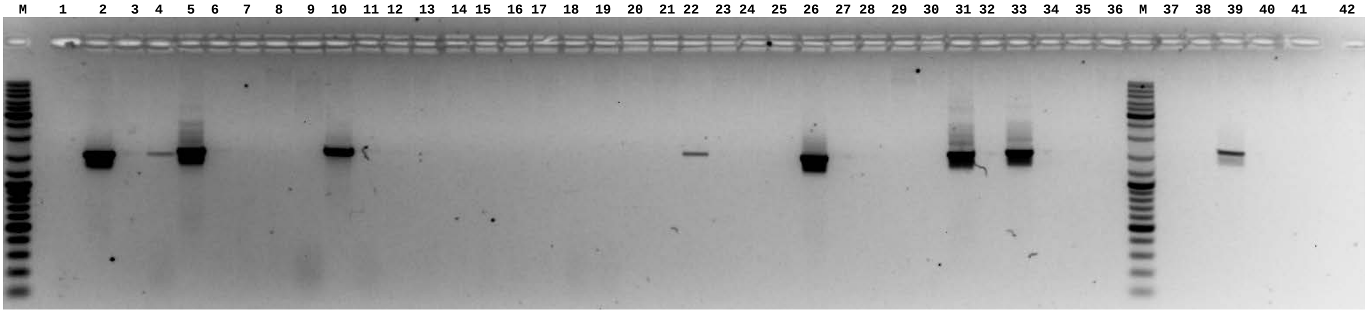


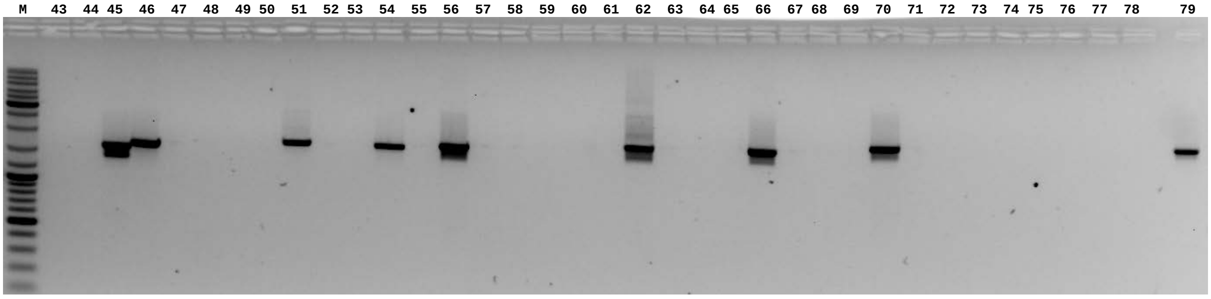


**DNA: 1-82 (**MT01-X1; 40-1-12-1**)**

**PCR**

1750 µl reaction mix contains 1120 µl MilliQ, 350 µl 5x Phusion HF Buffer, 35 µl dNTP (10 mM), 17 µl Thermo Scientific™ Phusion HS II Hot Start DNA Polymerase (2 U/µL), each 70 µl 5 µM primers (final **200 nM**).
Mix briefly and transfer 19 µl mix to PCR tube and add 1 µl DNA (30-60 ng/µl).

PCR amplification was carried out under the following conditions:
initial denaturation step at 98°C for **90 sec**, followed by **34x** amplifications at 98°C for **5 s**, **68°C for 30s**, **72°C for 20s**, followed by a final extension of 72°C for 1 min.

**20240410 18S-28S new universal primers test (5751-5756) new DNA human (positively psoriatic)**

*Test of the 5751/5756 pair on a freshly extracted panel of 46 stool DNA samples from psoriasis-positive donors, run in parallel with the 5728/5730 test of the previous day on overlapping templates, so that the two primer pairs can be compared on the same material.*

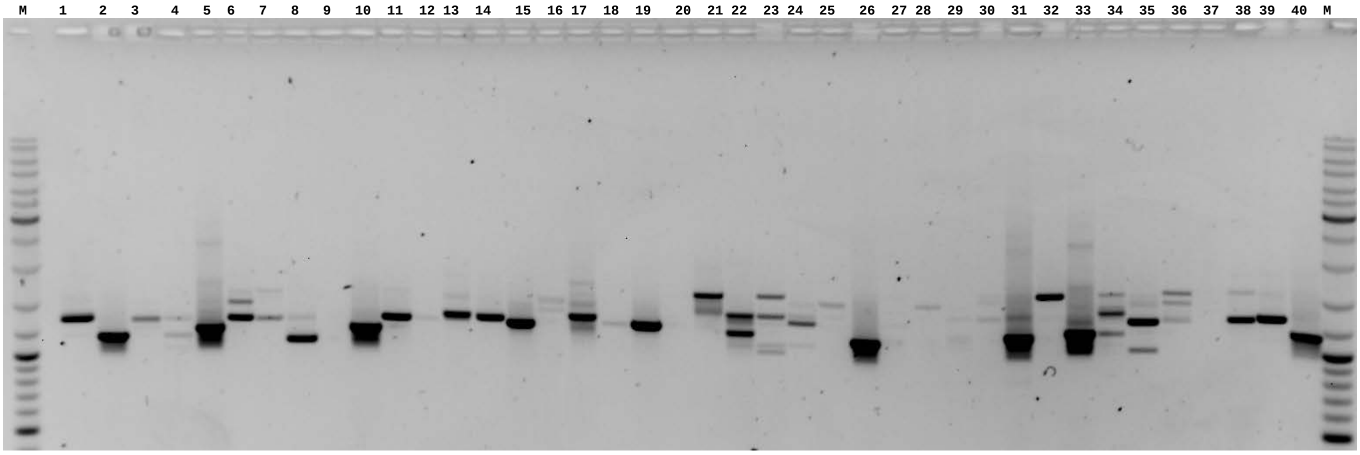


**DNA:**

| | | | | | | | | | | | |
|---|---|---|---|---|---|---|---|---|---|---|---|
| 1 | 2 | 3 | 4 | 5 | 6 | 7 | 8 | 9 | 10 | 11 | 12 |
| MT01 | MT02 | 05042023MT09 | MT5 | 05042024MT006T | 05042024MT007T | 8 | 9 | 10 | 11 | 12 | 13 |
| 13 | 14 | 15 | 16 | 17 | 18 | 19 | 20 | 21 | 22 | 23 | 24 |
| 14 | 16 | 17 | 18 | 19 | 20 | 33 | 1c-1 | 2c-1 | 3c-1 | 4c-1 | 5c-1 |
| 25 | 26 | 27 | 28 | 29 | 30 | 31 | 32 | 33 | 34 | 35 | 36 |
| 6c-1 | 7c-1 | 8c-1 | 9c-1 | 10c-1 | 11c-1 | 12c-1 | 13c-1 | 14c-1 | 15c-1 | 16c-1 | 17c-1 |
| 37 | 38 | 39 | 40 | 41 | 42 | 43 | 44 | 45 | 46 | | |
| 18c-1 | 19c-1 | X1 | 20c-1 | 21c-1 | 46c-1 | | | | | | |
| | | | | | | | | | | | |
| | | | | | | | | | | | |

**PCR**

1000 µl reaction mix contains 530 µl MilliQ, 200 µl 5x Phusion HF Buffer, 20 µl dNTP (10 mM), 10 µl Thermo Scientific™ Phusion HS II Hot Start DNA Polymerase (2 U/µL), each 200 µl 1 µM primers (final **200 nM**).
Mix briefly and transfer 21 µl mix to PCR tube and add 1 µl DNA (30-60 ng/µl).

PCR amplification was carried out under the following conditions:
initial denaturation step at 98°C for **90 sec**, followed by **31x** amplifications at 98°C for **5 s**, at **72°C for 60s**, followed by a final extension of 72°C for 1 min.

**20240409 18S-28S new universal primers test (5728-5730) new DNA human (positively psoriatic)**

*Test of the broad-range pair 5728/5730 on a freshly extracted panel of stool DNA from psoriasis-positive donors. This entry uses a two-step cycling programme with a combined annealing and elongation step at 72 °C for 90 s, in contrast to the three-step programme used in the March runs.*

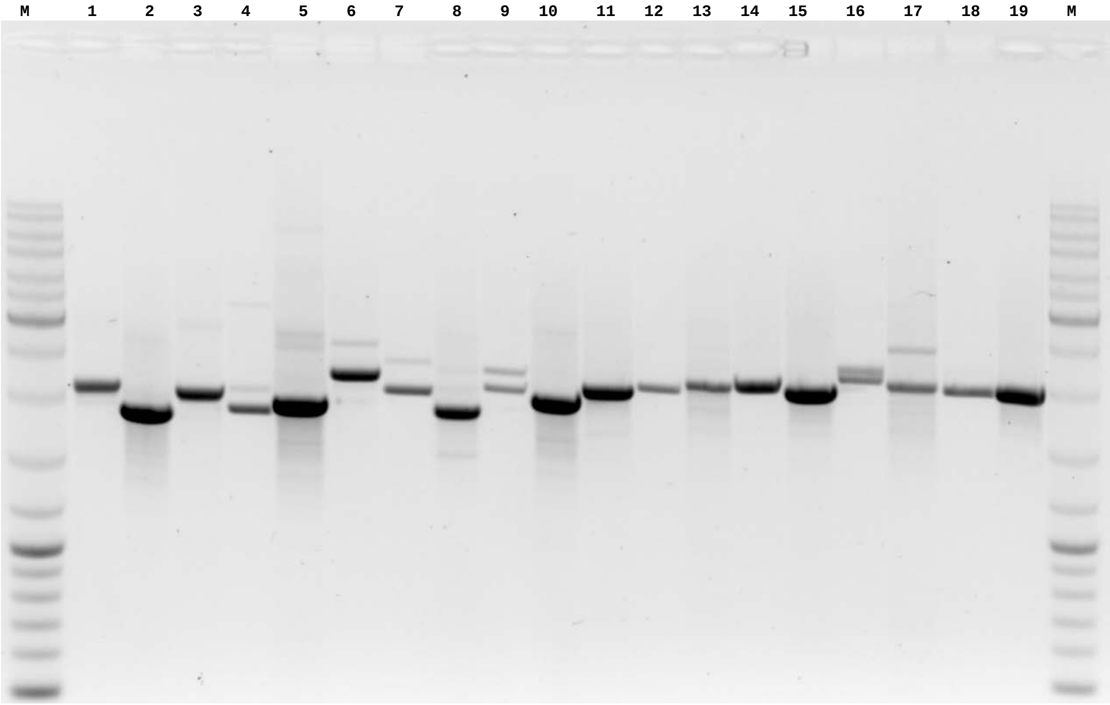


**DNA:**

| | | | | | | | | | | | |
|---|---|---|---|---|---|---|---|---|---|---|---|
| 1 | 2 | 3 | 4 | 5 | 6 | 7 | 8 | 9 | 10 | 11 | 12 |
| MT01 | MT02 | 05042023MT09 | MT5 | 05042024MT006T | 05042024MT007T | 8 | 9 | 10 | 11 | 12 | 13 |
| 13 | 14 | 15 | 16 | 17 | 18 | 19 | | | | | |
| 14 | 16 | 17 | 18 | 19 | 20 | 33 | | | | | |

## PCR

660 µl reaction mix contains 460 µl MilliQ, 132 µl 5x Phusion HF Buffer, 13.2 µl dNTP (10 mM), 6.6 µl Thermo Scientific™ Phusion HS II Hot Start DNA Polymerase (2 U/µL), each 26.4 µl 5 µM primers (final **200 nM**).
Mix briefly and transfer 29 µl mix to PCR tube and add 1 µl DNA (30-60 ng/µl).

PCR amplification was carried out under the following conditions:
initial denaturation step at 98°C for **90 sec**, followed by **35x** amplifications at 98°C for **5 s**, at **72°C for 90s**, followed by a final extension of 72°C for 1 min.

**20240404 18S-28S universal primers (5751-5758) test with DNA human faeces**

*Test of the 5751/5758 pair on sixteen extracts of the C series. This is the longest configuration examined, and the elongation time was extended to 120 s and the cycle number reduced to 30 accordingly.*

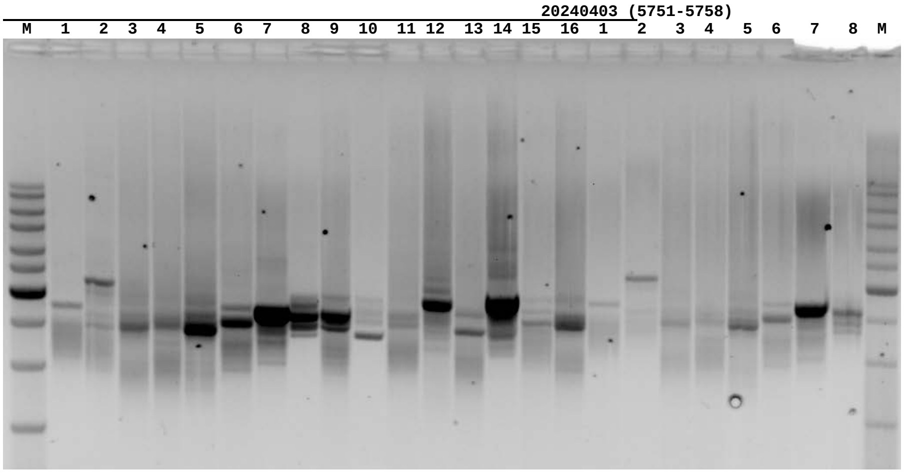


**DNA:**

| | | | | | | | | | | | |
|---|---|---|---|---|---|---|---|---|---|---|---|
| 1 | 2 | 3 | 4 | 5 | 6 | 7 | 8 | 9 | 10 | 11 | 12 |
| 1c-1 | 2c-1 | 3c-1 | 4c-1 | 5c-1 | 6c-1 | 7c-1 | 8c-1 | 9c-1 | 10c-1 | 11c-1 | 12c-1 |
| 13 | 14 | 15 | 16 | | | | | | | | |
| 13c-1 | 14c-1 | 15c-1 | 16c-1 | | | | | | | | |

**PCR**

400 µl reaction mix contains 212 µl MilliQ, 80 µl 5x Phusion HF Buffer, 8 µl dNTP (10 mM), 4 µl Thermo Scientific™ Phusion HS II Hot Start DNA Polymerase (2 U/µL), each 80 µl 1 µM primers (final **200 nM**).
Mix briefly and transfer 22 µl mix to PCR tube and add 1 µl DNA (30-60 ng/µl).

PCR amplification was carried out under the following conditions:
initial denaturation step at 98°C for **90 sec**, followed by **30x** amplifications at 98°C for **5 s**, at **72°C for 120s,** followed by a final extension of 72°C for 1 min.

**<u>20240403 18S-28S universal primers test with DNA human faeces</u>**

*Side-by-side comparison of five candidate reverse primers - 5756, 5754, 5759, 5758 and 5760 - each paired with the common forward primer 5751, on the same eight templates. Lanes are loaded in five blocks of eight in the order given in the heading, so that differences between the blocks reflect the reverse primer alone. This comparison, together with the repeat of 1 April, underlies the selection of the reverse primers reported in Table 1.*

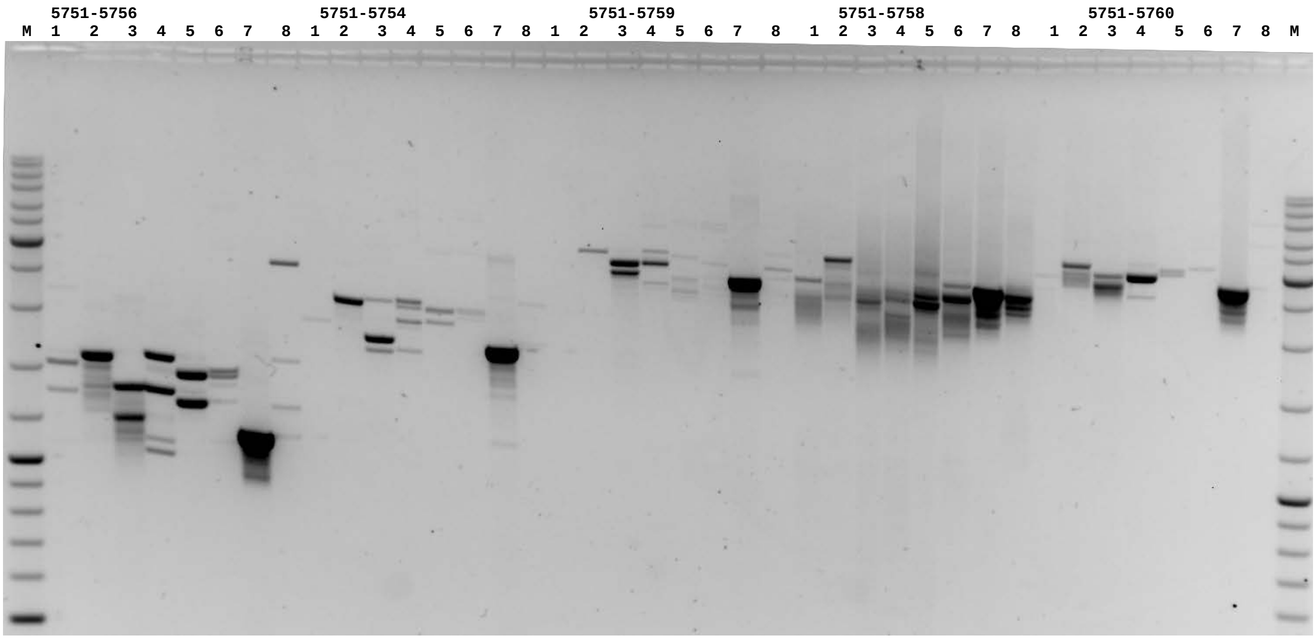


**DNA:**

| 1 | 2 | 3 | 4 | 5 | 6 | 7 | 8 |
|---|---|---|---|---|---|---|---|
| 1c-1 | 2c-1 | 3c-1 | 4c-1 | 5c-1 | 6c-1 | 7c-1 | 8c-1 |

## PCR

1000 µl reaction mix contains 520 µl MilliQ, 200 µl 5x Phusion HF Buffer, 20 µl dNTP (10 mM), 10 µl Thermo Scientific™ Phusion HS II Hot Start DNA Polymerase (2 U/µL), each 200 µl 1 µM primers (final **200 nM**).
Mix briefly and transfer 20 µl mix to PCR tube and add 1 µl DNA (30-60 ng/µl).

PCR amplification was carried out under the following conditions:
initial denaturation step at 98°C for **90 sec**, followed by **32x** amplifications at 98°C for **5 s**, at **72°C for 90s,** followed by a final extension of 72°C for 1 min.

**20240402 18S-28S new universal primers test (5753-5756) DNA human faeces**

*Application of the 5753/5756 pair to the full clinical panel of 44 stool DNA extracts, using a three-step programme with annealing at 68 °C. This is the alternative-pair counterpart to the 5728/5730 run on the same panel recorded under 3 February 2024.*

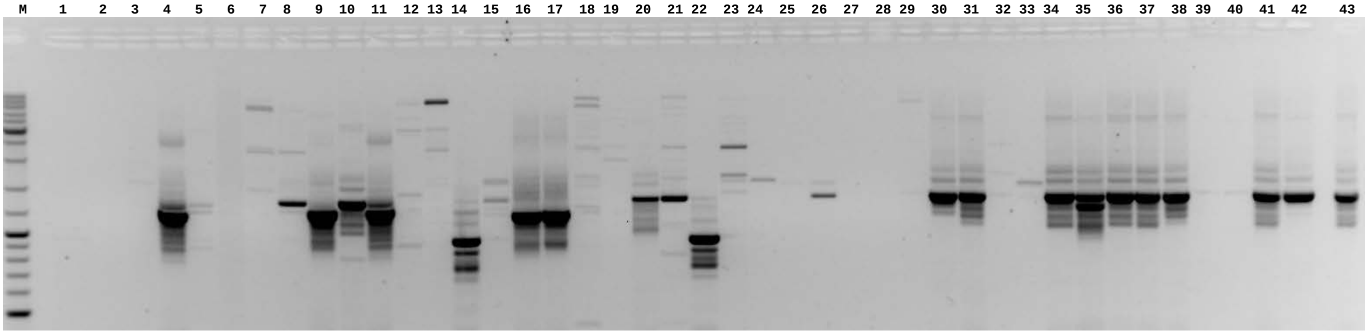


**DNA:**

| | | | | | | | | | | | |
|---|---|---|---|---|---|---|---|---|---|---|---|
| 1 | 2 | 3 | 4 | 5 | 6 | 7 | 8 | 9 | 10 | 11 | 12 |
| 005 | 007 | 012 | 016 | 019 | 0110 | w12008 | W12006 | W12014 | W12015 | W12016 | W12019 |
| 13 | 14 | 15 | 16 | 17 | 18 | 19 | 20 | 21 | 22 | 23 | 24 |
| W12020 | W12022 | TTK2110 | TTK2110 | TTK111 | TP005 | TP008 | TP010 | TP015 | TP022 | TP002 | TP003 |
| 25 | 26 | 27 | 28 | 29 | 30 | 31 | 32 | 33 | 34 | 35 | 36 |
| TП005 | TП006 | TП007 | TП008 | TП008 | TП009 | TП010 | TП012 | TП013 | TП014 | TП014 | TП015 |
| 37 | 38 | 39 | 40 | 41 | 42 | 43 | 44 | | | | |
| TП016 | TП018 | TП019 | TИ019 | TП021 | TП022 | TП023 | TП111 | | | | |

## PCR

1000 µl reaction mix contains 640 µl MilliQ, 200 µl 5x Phusion HF Buffer, 20 µl dNTP (10 mM), 10 µl Thermo Scientific™ Phusion HS II Hot Start DNA Polymerase (2 U/µL), each 2x40 µl 5 µM primers (final **200 nM**).
Mix briefly and transfer 20 µl mix to PCR tube and add 1 µl DNA (60-200 ng/µl).

PCR amplification was carried out under the following conditions:
initial denaturation step at 98°C for **90 sec**, followed by **32x** amplifications at 98°C for **5 s**, at **68°C for 30s, 72°C for 60s,** followed by a final extension of 72°C for 1 min.

<u>**20240401 18S-28S universal primers test with DNA human faeces**</u>

*Repeat of the five-way reverse-primer comparison (5751 paired in turn with 5756, 5754, 5759, 5758 and 5760) on the same eight templates, performed two days before the run of 3 April to confirm reproducibility of the pattern.*

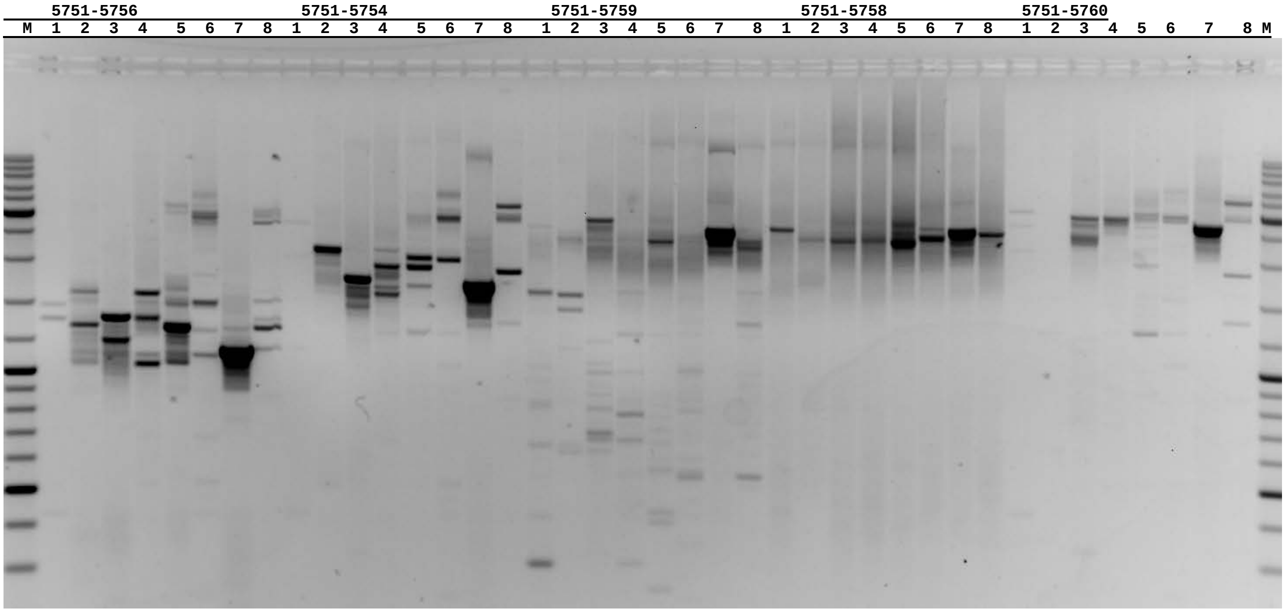


**DNA:**

| 1 | 2 | 3 | 4 | 5 | 6 | 7 | 8 |
|---|---|---|---|---|---|---|---|
| 1c-1 | 2c-1 | 3c-1 | 4c-1 | 5c-1 | 6c-1 | 7c-1 | 8c-1 |

**PCR**

1000 µl reaction mix contains 520 µl MilliQ, 200 µl 5x Phusion HF Buffer, 20 µl dNTP (10 mM), 10 µl Thermo Scientific™ Phusion HS II Hot Start DNA Polymerase (2 U/µL), each 200 µl 1 µM primers (final **200 nM**).
Mix briefly and transfer 20 µl mix to PCR tube and add 1 µl DNA (30-60 ng/µl).

PCR amplification was carried out under the following conditions:
initial denaturation step at 98°C for **90 sec**, followed by **34x** amplifications at 98°C for **5 s**, at **68°C for 60s, 72°C for 30s,** followed by a final extension of 72°C for 1 min.

**20240329 18S-28S new universal primers test (5753-5756) new DNA human faeces (control)**

*Control run of the 5753/5756 pair on 23 extracts of the C series, performed on the same template set as the 5728/5730 run of the previous day so that the two pairs can be compared directly.*

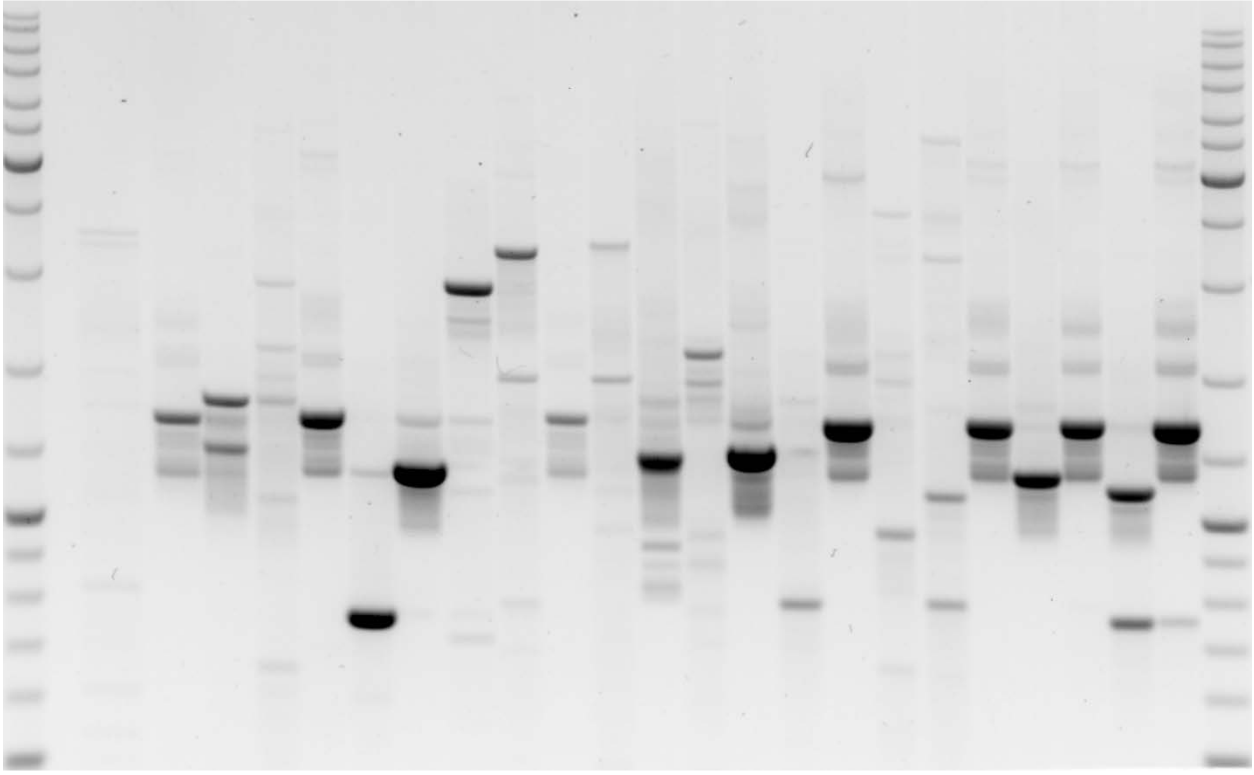


**DNA:**

| | | | | | | | | | | | |
|---|---|---|---|---|---|---|---|---|---|---|---|
| 1 | 2 | 3 | 4 | 5 | 6 | 7 | 8 | 9 | 10 | 11 | 12 |
| 1c-1 | 2c-1 | 3c-1 | 4c-1 | 5c-1 | 6c-1 | 7c-1 | 8c-1 | 9c-1 | 10c-1 | 11c-1 | 12c-1 |
| 13 | 14 | 15 | 16 | 17 | 18 | 19 | 20 | 21 | 22 | 23 | |
| 13c-1 | 14c-1 | 15c-1 | 16c-1 | 17c-1 | 18c-1 | 19c-1 | 20c-1 | 21c-1 | 46c-1 | X1 | |

## PCR

750 µl reaction mix contains 480 µl MilliQ, 150 µl 5x Phusion HF Buffer, 15 µl dNTP (10 mM), 7.5 µl Thermo Scientific™ Phusion HS II Hot Start DNA Polymerase (2 U/µL), each 2x 30 µl 5 µM primers (final **200 nM**).
Mix briefly and transfer 28.5 µl mix to PCR tube and add 1.5 µl DNA (30-60 ng/µl).

PCR amplification was carried out under the following conditions:
initial denaturation step at 98°C for **90 sec**, followed by **35x** amplifications at 98°C for **5 s**, at **68°C for 60s, 72°C for 30s,** followed by a final extension of 72°C for 1 min.

**20240328 18S-28S new universal primers test (5728-5730) new DNA human faeces (control)**

*Control run of the broad-range pair 5728/5730 on 23 extracts of the C series. Together with the 5753/5756 run of 29 March, this pair of experiments compares the two candidate combinations on identical material.*

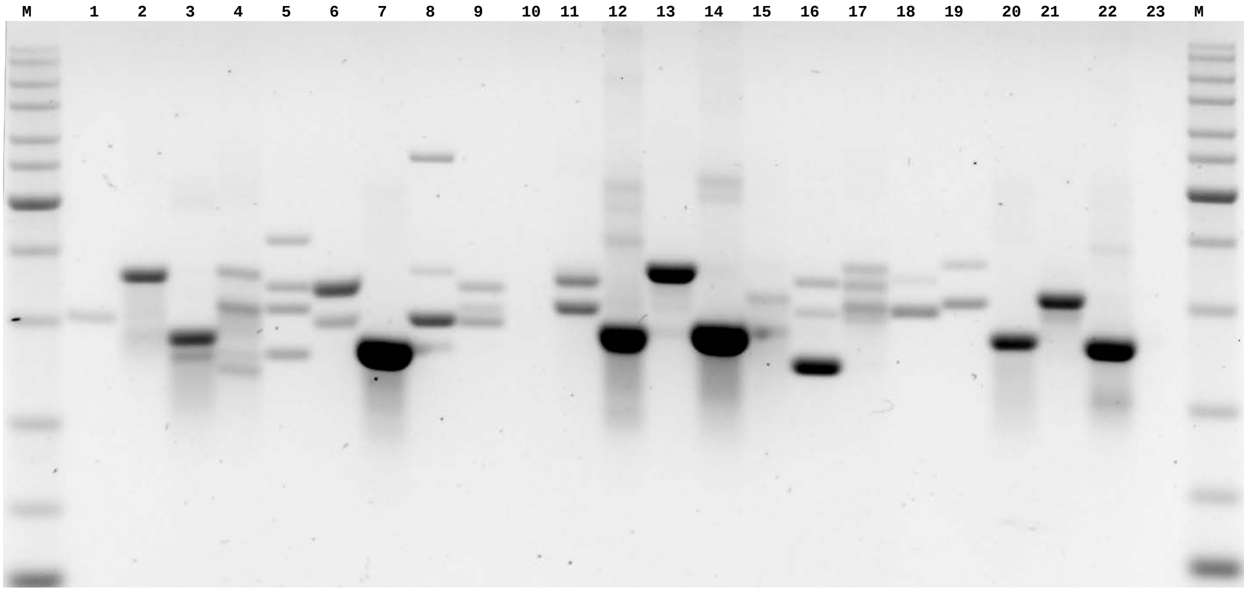


**DNA:**

| | | | | | | | | | | | |
|---|---|---|---|---|---|---|---|---|---|---|---|
| 1 | 2 | 3 | 4 | 5 | 6 | 7 | 8 | 9 | 10 | 11 | 12 |
| 1c-1 | 2c-1 | 3c-1 | 4c-1 | 5c-1 | 6c-1 | 7c-1 | 8c-1 | 9c-1 | 10c-1 | 11c-1 | 12c-1 |
| 13 | 14 | 15 | 16 | 17 | 18 | 19 | 20 | 21 | 22 | 23 | |
| 13c-1 | 14c-1 | 15c-1 | 16c-1 | 17c-1 | 18c-1 | 19c-1 | 20c-1 | 21c-1 | 46c-1 | X1 | |

**PCR**

750 µl reaction mix contains 510 µl MilliQ, 150 µl 5x Phusion HF Buffer, 15 µl dNTP (10 mM), 7.5 µl Thermo Scientific™ Phusion HS II Hot Start DNA Polymerase (2 U/µL), each 30 µl 5 µM primers (final **200 nM**).
Mix briefly and transfer 28.5 µl mix to PCR tube and add 1.5 µl DNA (30-60 ng/µl).

PCR amplification was carried out under the following conditions:
initial denaturation step at 98°C for **90 sec**, followed by **35x** amplifications at 98°C for **5 s**, at **72°C for 60s, 72°C for 30s,** followed by a final extension of 72°C for 1 min.

**20240327 18S-28S new universal primers test (5728-5730) new DNA human faeces for sequencing**

*Dedicated re-amplification, with the 5728/5730 pair, of nineteen templates selected from the screening run of the previous day, prepared specifically for band excision and capillary sequencing. Each lane yielded a single dominant product with only faint higher-molecular-weight secondary bands; the dominant bands were excised, gel-purified and sequenced, and the resulting consensus sequences are provided in PCR_amplicons.fasta.*

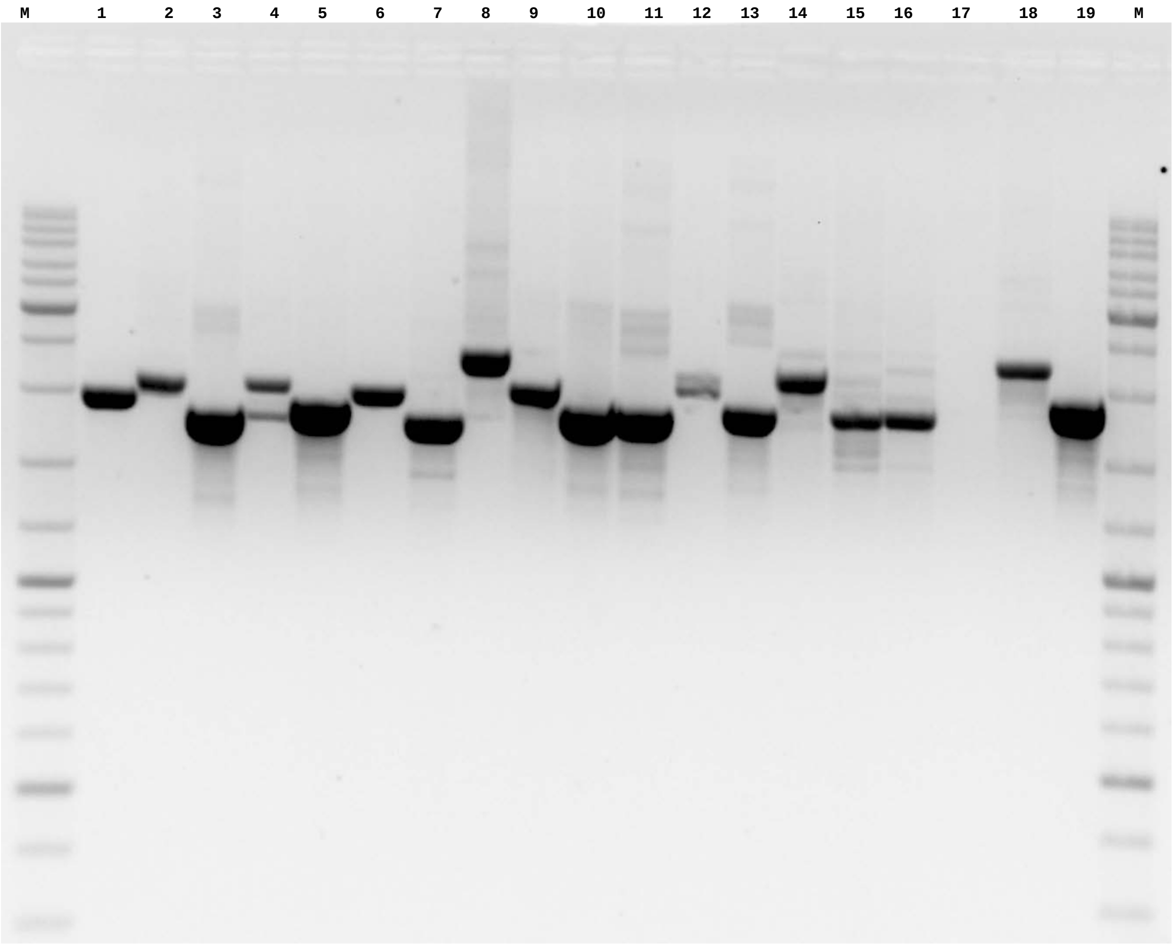


**DNA:**

```
1-8: TPC13 TPC7  1C    5C    6C    3C    43c-2 24C-1
8-16: 28C-1      49C-2 29C-1 36C-1 31c-1 32C-1 41C-1 40C-1
17-19: ~~33C-1~~     42c-1 38C-1
```

**PCR**

700 µl reaction mix contains 476 µl MilliQ, 140 µl 5x Phusion HF Buffer, 14 µl dNTP (10 mM), 7 µl Thermo Scientific™ Phusion HS II Hot Start DNA Polymerase (2 U/µL), each 28 µl 5 µM primers (final **200 nM**).
Mix briefly and transfer 28.5 µl mix to PCR tube and add 1.5 µl DNA (60-200 ng/µl).

PCR amplification was carried out under the following conditions:
initial denaturation step at 98°C for **90 sec**, followed by **35x** amplifications at 98°C for **5 s**, at **72°C for 60s, 72°C for 30s,** followed by a final extension of 72°C for 1 min.

## 20240326 18S-28S new universal primers test (5728-5730) new DNA human faeces (control)

*Screening run of the 5728/5730 pair across 43 stool DNA extracts of the TPC and C series, loaded on two gels. This is the run whose lane numbering is used in the sequence identifiers in PCR_amplicons.fasta: all sixteen templates that were subsequently sequenced appear on this gel, and their lane positions are quoted in the FASTA headers.*

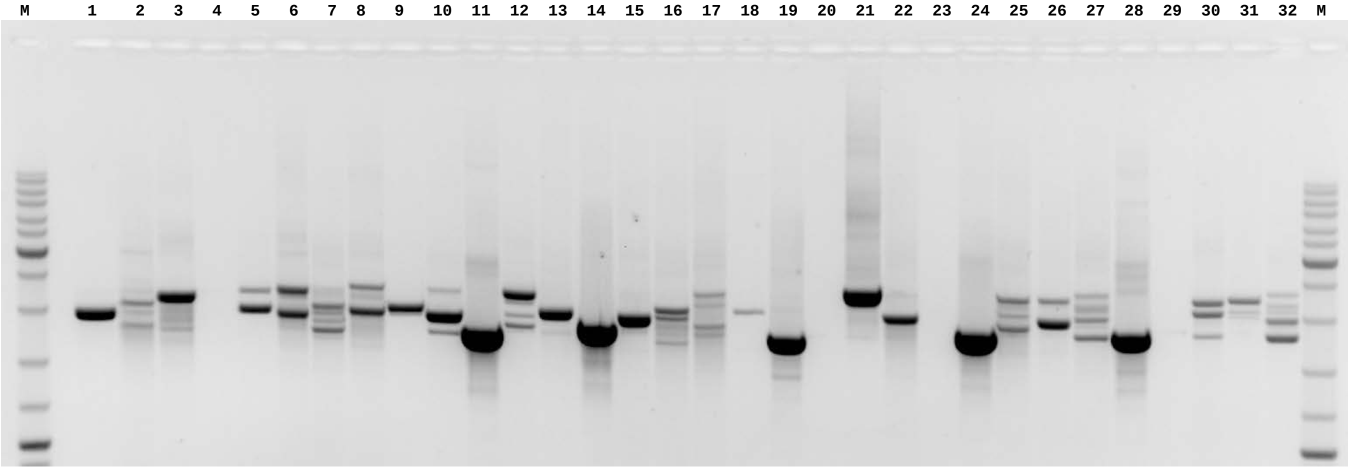


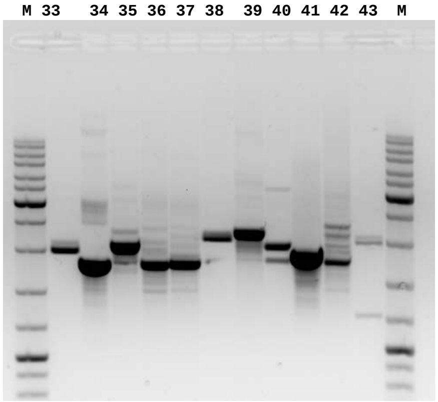


**DNA:**

| | | | | | | | | | | | |
|---|---|---|---|---|---|---|---|---|---|---|---|
| 1 | 2 | 3 | 4 | 5 | 6 | 7 | 8 | 9 | 10 | 11 | 12 |
| TPC13 | TPC12 | TPC8 | TPC10 | TPC2 | 8C | TPC11 | 7C | TPC7 | 4C | 1C | 2C |
| 13 | 14 | 15 | 16 | 17 | 18 | 19 | 20 | 21 | 22 | 23 | 24 |
| 5C | 6C | 3C | 48C-2 | 22C-1 | 47C-2 | 43C-2 | 44C-2 | 24C-1 | 28C-1 | 23C-1 | 49C-2 |
| 25 | 26 | 27 | 28 | 29 | 30 | 31 | 32 | 33 | 34 | 35 | 36 |
| 50C-2 | 45C-2 | 26C-1 | 29C-1 | 37C-1 | 30C-1 | 39C-1 | 34C-1 | 36C-1 | 31C-1 | 32C-1 | 41C-1 |
| 37 | 38 | 39 | 40 | 41 | 42 | 43 | | | | | |
| 40C-1 | 33C-1 | 42C-1 | 35C-1 | 38C-1 | 25C-1 | 27C-1 | | | | | |

### PCR

1400 µl reaction mix contains 970 µl MilliQ, 280 µl 5x Phusion HF Buffer, 28 µl dNTP (10 mM), 14 µl Thermo Scientific™ Phusion HS II Hot Start DNA Polymerase (2 U/µL), each 56 µl 5 µM primers (final **200 nM**).
Mix briefly and transfer 28 µl mix to PCR tube and add 1.5 µl DNA (60-200 ng/µl).

PCR amplification was carried out under the following conditions:
initial denaturation step at 98°C for **90 sec**, followed by **35x** amplifications at 98°C for **5 s**, at **72°C for 60s, 72°C for 30s,** followed by a final extension of 72°C for 1 min.

## 202400203 18S-28S new universal primers test (5728-5730) DNA human faeces

*Additional five cycles applied to the reaction plate of the preceding entry, in order to increase product yield for templates that had given weak signal. The template panel and lane assignments are unchanged; only the supplementary cycling programme is recorded here.*

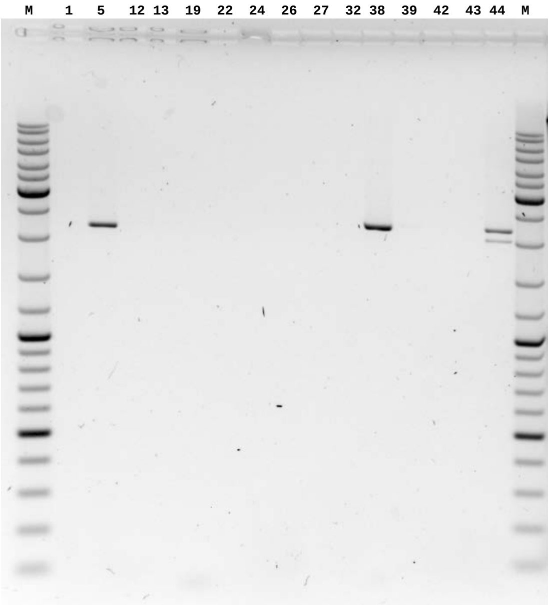


**DNA:**

| | | | | | | | | | | | |
|---|---|---|---|---|---|---|---|---|---|---|---|
| 1 | 2 | 3 | 4 | 5 | 6 | 7 | 8 | 9 | 10 | 11 | 12 |
| 005 | 007 | 012 | 016 | 019 | 0110 | w12008 | W12006 | W12014 | W12015 | W12016 | W12019 |
| 13 | 14 | 15 | 16 | 17 | 18 | 19 | 20 | 21 | 22 | 23 | 24 |
| W12020 | W12022 | TTK2110 | TTK2110 | TTK111 | TP005 | TP008 | TP010 | TP015 | TP022 | TP002 | TP003 |
| 25 | 26 | 27 | 28 | 29 | 30 | 31 | 32 | 33 | 34 | 35 | 36 |
| ТП005 | ТП006 | ТП007 | ТП008 | ТП008 | ТП009 | ТП010 | ТП012 | ТП013 | ТП014 | ТП014 | ТП015 |
| 37 | 38 | 39 | 40 | 41 | 42 | 43 | 44 | | | | |
| ТП016 | ТП018 | ТП019 | ТИ019 | ТП021 | ТП022 | ТП023 | ТП111 | | | | |

## PCR: 202400203

Additional 5 PCR cycles:initial denaturation step at 98°C for **30 sec**, followed by **5x** amplifications at 98°C for **5 s**, at **72°C for 60s, 72°C for 60s,** followed by a final extension of 72°C for 1 min.

**202400203 18S-28S new universal primers test (5728-5730) DNA human faeces**

*First application of the broad-range pair 5728/5730 to the clinical panel of 44 stool DNA extracts, spanning the 005, W12, TTK, TP and ТП identifier series. This is the earliest experiment in the record and establishes the amplification conditions used throughout the March runs.*

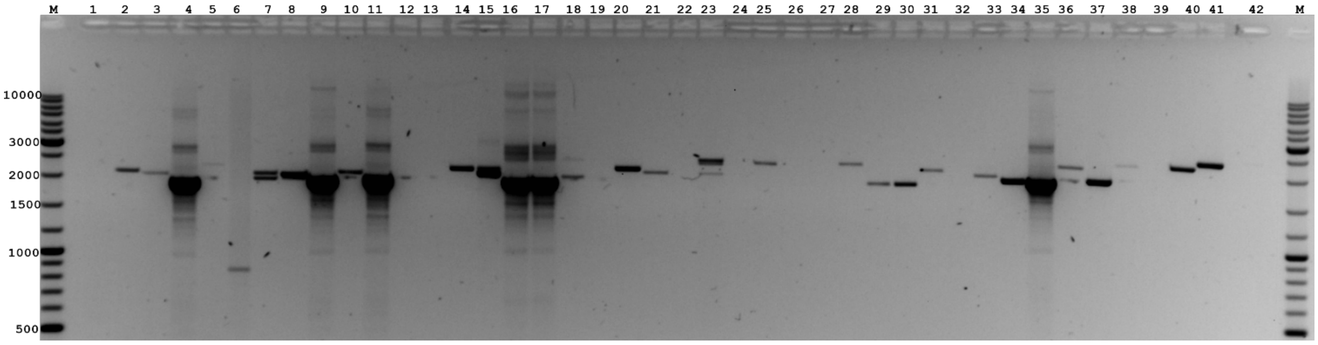


**DNA:**

| | | | | | | | | | | | |
|---|---|---|---|---|---|---|---|---|---|---|---|
| 1 | 2 | 3 | 4 | 5 | 6 | 7 | 8 | 9 | 10 | 11 | 12 |
| 005 | 007 | 012 | 016 | 019 | 0110 | w12008 | W12006 | W12014 | W12015 | W12016 | W12019 |
| 13 | 14 | 15 | 16 | 17 | 18 | 19 | 20 | 21 | 22 | 23 | 24 |
| W12020 | W12022 | TTK2110 | TTK2110 | TTK111 | TP005 | TP008 | TP010 | TP015 | TP022 | TP002 | TP003 |
| 25 | 26 | 27 | 28 | 29 | 30 | 31 | 32 | 33 | 34 | 35 | 36 |
| ТП005 | ТП006 | ТП007 | ТП008 | ТП008 | ТП009 | ТП010 | ТП012 | ТП013 | ТП014 | ТП014 | ТП015 |
| 37 | 38 | 39 | 40 | 41 | 42 | 43 | 44 | | | | |
| ТП016 | ТП018 | ТП019 | ТИ019 | ТП021 | ТП022 | ТП023 | ТП111 | | | | |

**PCR**

1400 µl reaction mix contains: 970 µl MilliQ, 280 µl 5x Phusion HF Buffer, 28 µl dNTP (10 mM), 14 µl Thermo Scientific™ Phusion HS II Hot Start DNA Polymerase (2 U/µL), each 56 µl 5 µM primers (final **200 nM**).
Mix briefly and transfer 28 µl mix to PCR tube and add 1.5 µl DNA (60-200 ng/µl).

PCR amplification was carried out under the following conditions:
initial denaturation step at 98°C for **90 sec**, followed by **35x** amplifications at 98°C for **5 s**, at **72°C for 60s, 72°C for 60s,** followed by a final extension of 72°C for 1 min.

**20240125 18S-28S new universal primers test (5765/5656-5751) for plant**

*Head-to-head comparison of the plant-selective reverse primer 5765 with the broad-range reverse primer 5756, each paired with the same forward primer 5751 and applied to the same twelve plant DNA extracts. Lanes 1-12 carry the 5765/5751 products and lanes 13-24 the 5756/5751 products, so differences between the two blocks reflect the reverse primer alone. The 5765/5751 combination produced a single product of consistent size in all twelve lanes, whereas 5756/5751 produced multiple products of variable size in most lanes. This contrast is the primary experimental evidence for the greater specificity of 5765 and underlies its designation as a Viridiplantae-selective primer in Table 1 of the manuscript.*

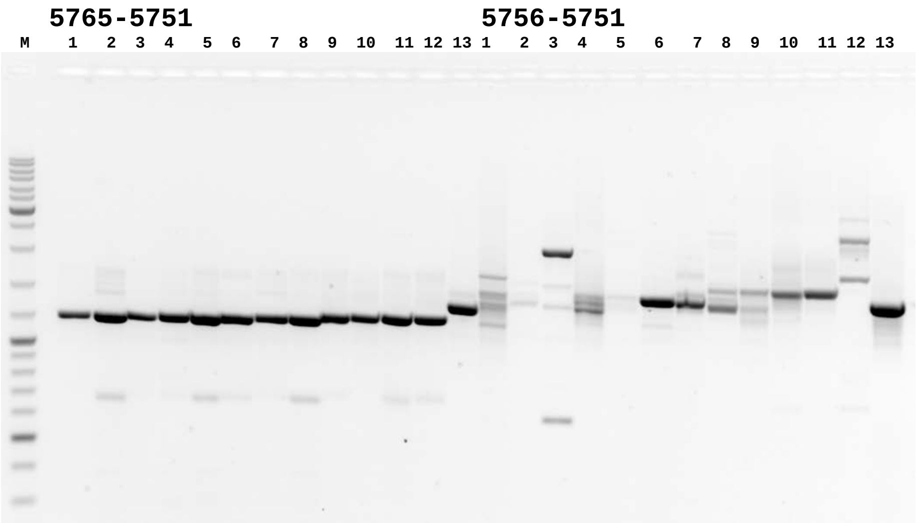


01-12: 5765-5751
13-24: 5756-5751

**Plants:**

| | |
|---|---|
| 1 | Gagea altaica |
| 2 | Gagea ovl |
| 3 | Gagea sarisulash |
| 4 | Gagea ilzechchstanin |
| 5 | Gagea altaica |
| 6 | Gagea sanisucrsis |
| 7 | Gagea lasinalata |
| 8 | Gagea altaica |
| 9 | Tulipa biebersteiniana |
| 10 | Tulipa patens |
| 11 | Tulipa auliecolica |
| 12 | Tulipa turgaica (20) |
| 13 | xx |

**PCR**

700 µl reaction mix contains: 455 µl MilliQ, 140 µl 5x Phusion HF Buffer, 14 µl dNTP (10 mM), 7 µl Thermo Scientific™ Phusion HS II Hot Start DNA Polymerase (2 U/µL), each 28 µl 5 µM primers (final **200 nM**).
2x mix add 14 µl 5 µM primers (final **200 nM**)
Mix briefly and transfer 25 µl mix to PCR tube and add 1.1 µl DNA

PCR amplification was carried out under the following conditions:
initial denaturation step at 98°C for **90 sec**, followed by **31x** amplifications at 98°C for **10 s**, at **68°C** **for 60s, 72°C for 30s,** followed by a final extension of 72°C for 1 min.

**20240124 18S-28S new universal primers test (5765-5751) for plant**

*First test of the Viridiplantae-selective configuration. The plant-specific 28S reverse primer 5765 was paired with the broad-range 18S forward primer 5751 and applied to thirteen purified plant DNA extracts from two Liliaceae genera (Gagea and Tulipa), to a pooled DNA mixture, and to environmental DNA samples (one CTAB extract and seventeen extracts of the Caspian series), the last representing mixed-template material in which plant, fungal and animal sequences co-occur. The purified plant templates yielded a strong product of uniform size in every lane. Under these conditions the environmental samples gave a visible product in only a few lanes and none in the majority, so this run establishes that the pairing amplifies plant templates efficiently but does not by itself demonstrate selective recovery of plant sequences from mixed environmental DNA.*

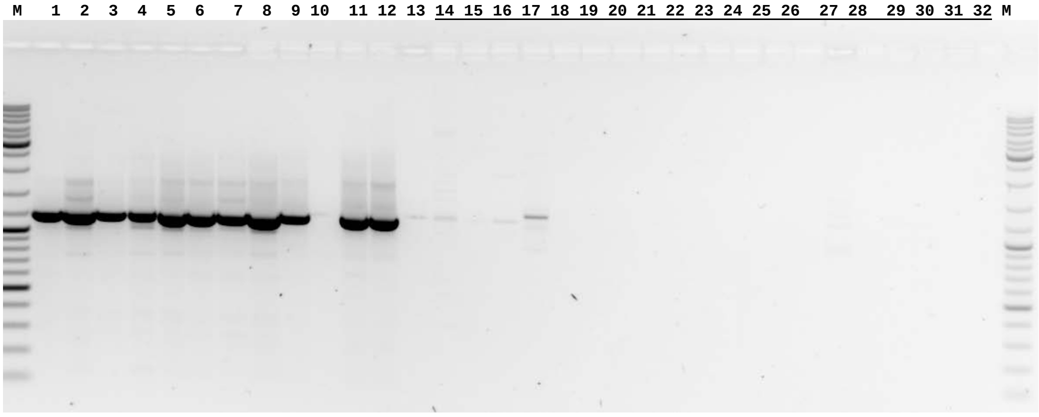


**Plants:**

| | |
|---|---|
| 1 | Gagea altaica |
| 2 | Gagea ovl |
| 3 | Gagea sarisulash |
| 4 | Gagea ilzechchstanin |
| 5 | Gagea altaica |
| 6 | Gagea sanisucrsis |
| 7 | Gagea lasinalata |
| 8 | Gagea altaica |
| 9 | Tulipa biebersteiniana |
| 10 | Tulipa patens |
| 11 | Tulipa auliecolica |
| 12 | Tulipa turgaica (20) |
| 13 | Tulipa buhscani |
| 14 | DNA mix |
| 15 | eDNA CTAB |
| 16-32 | eDNA_Caspian Sea |

**PCR**

900 µl reaction mix contains: 585 µl MilliQ, 180 µl 5x Phusion HF Buffer, 18 µl dNTP (10 mM), 9 µl Thermo Scientific™ Phusion HS II Hot Start DNA Polymerase (2 U/µL), each 36 µl 5 µM primers 5765 -5751 (final **200 nM**).

Mix briefly and transfer 25 µl mix to PCR tube and add 1 µl DNA

PCR amplification was carried out under the following conditions:
initial denaturation step at 98°C for **90 sec**, followed by **32x** amplifications at 98°C for **10 s**, at **68°C** **for 60s, 72°C for 30s,** followed by a final extension of 72°C for 1 min.
